# CRX directs photoreceptor differentiation by accelerating chromatin remodeling at specific target sites

**DOI:** 10.1186/s13072-018-0212-2

**Published:** 2018-08-01

**Authors:** Philip A. Ruzycki, Xiaodong Zhang, Shiming Chen

**Affiliations:** 1 Department of Ophthalmology and Visual Sciences, Washington University School of Medicine, St. Louis, MO USA; 2 Department of Developmental Biology, Washington University School of Medicine, St. Louis, MO USA; 3 Molecular Genetics and Genomics Graduate Program, Division of Biology and Biomedical Sciences, Washington University School of Medicine, St. Louis, MO USA

**Keywords:** Retinal gene expression, Photoreceptor development, *Crx* deficiency, Epigenetic regulation

## Abstract

**Background:**

Recent technological advances have delivered the genome-wide targets of many important transcription factors (TFs). However, increasing evidence suggests that not all target sites mediate regulatory function, raising the questions of how to determine which sites are active, what are the epigenetic consequences of TF binding at these sites, and how the specificity is coded. To address these questions, we focused on CRX, a disease-associated homeodomain TF required for photoreceptor gene expression and development. Since CRX binds more than 6000 sites across the genome in the retina, we profiled chromatin landscape changes at each binding site during normal development and in the absence of CRX and interpreted the results by thorough investigation of other epigenomic datasets and sequence features.

**Results:**

CRX is required for chromatin remodeling at only a subset of its binding sites, which undergo retina or neuronal specific activation during photoreceptor differentiation. Genes near these “CRX Dependent” sites code for proteins important for photoreceptor physiology and function, and their transcription is significantly reduced in *Crx* deficient retinas. In addition, the nucleotide and motif content distinguish these CRX Dependent sites from other CRX-bound sites.

**Conclusions:**

Together, our results suggest that CRX acts only at select, uniquely-coded binding sites to accelerate chromatin remodeling during photoreceptor differentiation. This study emphasizes the importance of connecting TF binding with its functional consequences and provides a framework for making such a connection using comparative analyses of available genomic datasets. Finally, this study prioritizes sets of non-coding DNA sites for future functional interrogation and identification of mutations associated with retinal disease.

**Electronic supplementary material:**

The online version of this article (10.1186/s13072-018-0212-2) contains supplementary material, which is available to authorized users.

## Background

Development and maintenance of each cell type in our body requires precisely regulated gene expression, where a set of genes required for specific cellular structure and function is activated, but other irrelevant genes are silenced. This is directed by transcription factor (TF) networks and their target DNA elements across the genome. Recent technological advances have delivered the genome-wide binding sites of many TFs essential for a wide range of developmental processes and cell types. However, our knowledge about how specific TFs work is still quite limited. For instance, a TF will not bind every instance of its target DNA motif in the genome. Furthermore, each binding occurrence of the same TF may not have the same functional consequence or relevance. In this study, we sought to address these questions using the retinal and disease relevant homeodomain TF CRX as a model.

The retina is the highly specialized portion of the central nervous system responsible for initiating and processing visual signals before they are transmitted to the brain. The retina consists of six major classes of neurons and one of glia [1–4]. Rods and cones are the two types of photoreceptors responsible for the initial conversion of a photon of light into an electrical signal. Mouse retinas are rod dominant; rods constitute 80% of the retinal cells, while cones comprise only 2% [1, 5]. Retinal neurogenesis follows a stereotyped developmental program with specific cell types born in overlapping waves [6]. In mice, rods are born over a long window in time that peaks at postnatal day 0 (P0) and continues until P2 [6]. Post-mitotic rod precursors undergo differentiation over an extended 2-week period, during which the cells establish a rod-specific gene expression profile, develop unique subcellular structures, and eventually can perform phototransduction.

Precisely regulated gene expression is essential for rod structural/functional development and survival, as even subtle perturbations can result in blinding diseases [7, 8]. Rod gene expression is tightly regulated by a number of transcription factors (TFs), acting in a cascade during development [(Reviewed by Swaroop et al. [9]). The homeodomain (HD) TF OTX2 specifies the photoreceptor lineage by turning on the expression of cone rod homeobox (CRX) and its downstream TFs. CRX is an OTX-like HD TF, whose expression coincides with the final mitotic event in rod and cone photoreceptors and is maintained into adulthood [10, 11]. CRX binds to the promoter of rod/cone genes and activates their expression via its transactivation domain [10, 12]. Two rod-specific TFs, NRL and NR2E3, act with CRX to direct rod differentiation by activating rod and silencing cone genes [9, 13–15]. General TFs involved in chromatin remodeling, including MEF2D, CBP/P300, and the STAGA complex, are also part of the CRX regulatory network [16–18]. Together, these factors properly establish the rod epigenome and transcriptome.

CRX is essential for photoreceptor differentiation and functional development. A *Crx* null mutation (*Crx*−*/*−) produces a recessive phenotype in the mouse retina where the immature photoreceptor cells fail to differentiate and begin to degenerate at 4 weeks of age [19]. Mutations in human CRX have been associated with dominant blinding retinopathies with varying severity and etiology (Reviewed by Tran and Chen [20]). Interestingly, in the corresponding mouse models, distinct *Crx* mutations all affect the expression of a common set of genes, but the degree of dysregulation correlates with phenotype severity [7]. The mechanism for this mis-regulation remains to be determined, but a better understanding of CRX’s mechanism of action during photoreceptor development would provide insights into the pathogenicity of human CRX mutations.

Chromatin immunoprecipitation with high-throughput sequencing (ChIP-seq) for CRX was performed in the adult mouse retina [21]. Binding sites were enriched for a HD binding motif, and authors noted that many known photoreceptor-specific genes were near binding sites. These same genes generally lose expression in *Crx* mutant retinas [22]. However, while these analyses suggest that those genes are directly controlled by proximal CRX-binding sites, they fail to explain the function of the many other CRX-bound sites that are not near affected genes.

Previous experiments have also investigated the activity of CRX using plasmid-based assays. Luciferase reporter assays in heterologous systems have been employed to map the activation domain, demonstrate the synergy between CRX and NRL, and determine the functional effect of CRX mutations [12, 22–24]. Plasmid-based systems have been adapted to report the activity of enhancers *in vivo* using GFP [21, 25, 26], but these experiments are limited in scope as each construct must be tested individually. Recent technological advances have overcome these throughput limitations. Massively Parallel Reporter Assays (MPRAs) performed in the retina confirmed that hundreds of CRX-bound regulatory elements positively regulate transcription, while unbound regions did not [27]. Additional experiments suggested that motif affinity directly contributed to this regulatory potential [28]. However, it remains to be seen if these MPRA findings can be translated into the genomic/chromatin context *in vivo*.

Multiple experiments have sought to understand normal epigenetic development of rods and cones [29–33]. Dramatic developmental remodeling was described by ChIP-seq of histones and regulatory proteins, and by profiling DNA methylation [30]. Other studies have profiled DNA accessibility through development or compared mature rods versus cones [29, 31–33]. While these studies all inferred the relevance of CRX at the many regulatory elements to which it binds, none expressly tested this *in vivo*. Other recent efforts have profiled epigenetic changes in diseased human retina [34]. Most importantly, this study identified a loss of epigenetic activity at retinal sites with the CRX motif. However, additional experimental tests will be necessary to establish a causative role of CRX in the disease.

To bridge this gap in the understanding of TF binding versus regulatory potential, here we use ATAC-seq to show that CRX is only responsible for the epigenomic rearrangement of a subset of its binding sites. These “Dependent” sites reside within a variety of chromatin environments and are highly correlated with the genes affected in mutant retinas. By applying this technique, we clarify the role of this important TF in retinal development and disease and provide a model for future studies of TFs essential to the development of other cell types.

## Methods

### Animals

Wild-type and *Crx*−*/*− mice (kindly provided by Dr. Connie Cepko at Harvard University) used for experiments were backcrossed (> 10 generations) to *C57BL/6* *J* mice obtained from the Jackson Laboratory (Bar Harbor, Maine; Stock # 000664).

### ATAC-seq library prep and sequencing

ATAC-seq was performed as published in Buenrostro et al. [35]. Briefly, retinas were dissected from P14 *WT* and *Crx*−*/*− mice and washed in PBS. Tissue was dissociated at 37C using 2% collagenase in TESCA buffer for 13 min and the reaction stopped by the addition of 2X volume of DMEM + 10% FBS. DNase I (0.5 Units; Roche, Basel, Switzerland) was added for the final 3 min to minimize clumping of cells. Cells were counted with hemocytometer and 50,000 re-suspended in TD buffer for a 1 h incubation with TDE1 (Nextera DNA Library Prep Kit; Illumina, San Diego, CA) at 37C. Remaining library prep was performed as published. Libraries were pooled and sequenced using the Illumina 2500.

### Chromatin Immunoprecipitation

Chromatin immunoprecipitation (ChIP) assay was performed as previously described [22]. In summary, 6 pooled P14 *C57BL/6* *J* wild-type or *Crx*−*/*− mouse retinas per sample were dissected and chromatin was cross-linked with 1% formaldehyde in PBS for 10 min at room temperature. Cross-linked cells were lysed and fragmented by sonication. Chromatin fragments were immunoprecipitated with the antibodies to H3K27ac (Abcam, Cambridge, UK; ab4729) and H3K4me3 (Millipore Sigma, Burlington, MA; 07-473), or normal rabbit/mouse IgG (Santa Cruz Biotechnology, Dallas, TX) bound to Protein A beads (Millipore, 16-125) or A/G beads (Santa Cruz Biotechnology). After extensive washing, the immunoprecipitated chromatin was eluted, heated to 67 °C to reverse the cross-links, and the DNA-purified by ethanol precipitation. Libraries were prepared using the DNA SMART ChIP-Seq Kit (Clonetech, Mountain View, CA). 10 ng of ChIP DNA was used as input for each sample.

### Mapping of ATAC-seq and ChIP-seq data

Libraries were de-multiplexed according to barcodes inserted in the P7 adaptor and mapped to mm9 using Novoalign (V3.04.06). Alignments were cleaned using Samtools (V1.3.1); duplicate and reads mapping to mitochondrial genome were removed. All other processing for visualization in IGV was performed using Samtools and BEDTools (V2.24.0). For visualization, bedtools slop function was used to extend reads 300 bp.

### Peak calling and genotype comparison

Peak calling of ChIP [histone and CRX] and ATAC-seq data was performed using MACS2 (V2.1.0.20140616). Peak calling was performed on replicate samples independently. Only peaks that replicated in both samples were kept, by comparing peak files using the bedtools intersect function. Intersecting peaks were merged using bedtools merge function and analyzed for number of reads within each library using bedtools coverage. Statistical comparison of ATAC-seq data was performed using EdgeR (V3.18.1). Peaks that did not pass the filter criteria of counts per million (CPM) ≥ 5 in at least 2 samples were removed prior to the analysis. Filtered count data were normalized by the EdgeR default normalization method, TMM, and differential analysis was performed by the exact test. P-values were subjected to Bonferroni and Hochberg multiple testing correction to include false discovery rate (FDR). Peaks deemed to increase or decrease in the *Crx*−*/*− retina were those with a fold-change ≥ 2 and FDR < 0.05.

Replicate histone experimental datasets were normalized to RPM before subtraction of input signal within peak regions. These values were averaged between replicates for comparison of *WT* and *Crx*−*/*− retina samples. Analysis and quantification of ATAC-seq and ChIP-seq data are provided in Additional file 1.

### Detection of overlapping ChIP, ATAC, and ChromHMM location

All co-localization detection of genome-wide datasets was performed using the bedtools intersect function. ChromHMM bed files were graciously provided by Dr. Issam Aldiri, St. Jude’s Children’s Research Hospital.

### Read coverage epigenetic analysis

Heatmaps and line graphs depicting epigenetic data were generated using the UNIX software package HOMER (V4.7) [36]. Heatmaps were generated by importing the HOMER-generated counts back into R and visualized by the heatmap.2 function in the gplots (V3.0.1) R package. Line graphs plotting average signal were computed from this data in R. Statistical comparison and plotting of data was performed using GraphPad Prism software (V7.0b).

### Motif calling

Known motif analysis was performed using Transcription Factor Affinity Prediction (TRAP) web Tools [37]. Analysis was performed on Jaspar vertebrate matrix file, with mouse promoter background model, and multiple test correction was Benjamini–Hochberg. Heatmap visualizes the −log(10)-converted corrected p-value of each TF motif. *De novo* motif analysis was performed using the HOMER findmotifsgenome tool (V4.7).

### BEEML calculation of affinity

Analyses were performed as in White et al. [28]. Custom scripts to calculate TF affinity were graciously provided by authors. Statistical comparisons of results were performed in R.

### Nucleotide density and motif density

All analyses were centered on the CRX ChIP peak and utilized the HOMER annotatepeaks function. Matrix outputs from de novo motif analysis were tested for motif density using the HOMER annotatepeaks function in 20 bp windows.

### Conservation

All analyses were centered on the CRX ChIP peak and utilized data downloaded from UCSC genome browser (phastCons30way Vertebrate Conservation). Plotted data represent mean of a running 20-bp window across the peak region.

### Gene ontology (GO) analysis

Gene ontology (GO) analyses were performed using the GREAT tool (Ver 3.0) [38]. Peak files used were centered on the CRX-binding site, and associations were with the settings “single nearest gene” and no limit on distance. These same associations were used as the basis of regulatory site to gene association for RNA-seq comparisons.

### RNA-seq

RNA-seq data were obtained from GSE52006. Data were analyzed as described previously in Ruzycki *et al* (2016) [7]. Briefly, 1 × 42 bp reads were aligned to the mouse genome (version mm9) with the sequence aligner TopHat2 (version v2.0.5) using the following parameters: -a 5 -m 1 -i 10 -I 500000 -r 100 –p 4 –microexon-search –no-coverage-search -× 20 –segment-length 25. Dependencies included Bowtie (v0.12.8) and Samtools (v0.1.18). Bedgraph files were generated using BEDTools (v2.23.0) and visualized using IGV (V2.3.20). The HTSeq package (version 0.6.1p1) was used to assign aligned reads to the gene annotation reference track (UCSC Genes Track, UCSC Table Browser, NCBI37/mm9, accessed July 16, 2014). This generated a raw read count per gene which was used in EdgeR [39] for detecting differentially expressed genes. For each of the genotype comparisons, genes that did not pass the filter criteria of counts per million (CPM) ≥ 5 in all replicates of at least one comparison Group were removed prior to the analysis. Filtered count data were normalized by the EdgeR default normalization method, TMM, and differential expression analysis for each of the comparison groups was performed by the exact test. P-values were subjected to Bonferroni and Hochberg multiple testing correction to include false discovery rate (FDR). Downstream analysis was performed using custom Perl and R scripts.

## Results

### CRX binds ATAC-sensitive regulatory sites

**Fig. 1:**
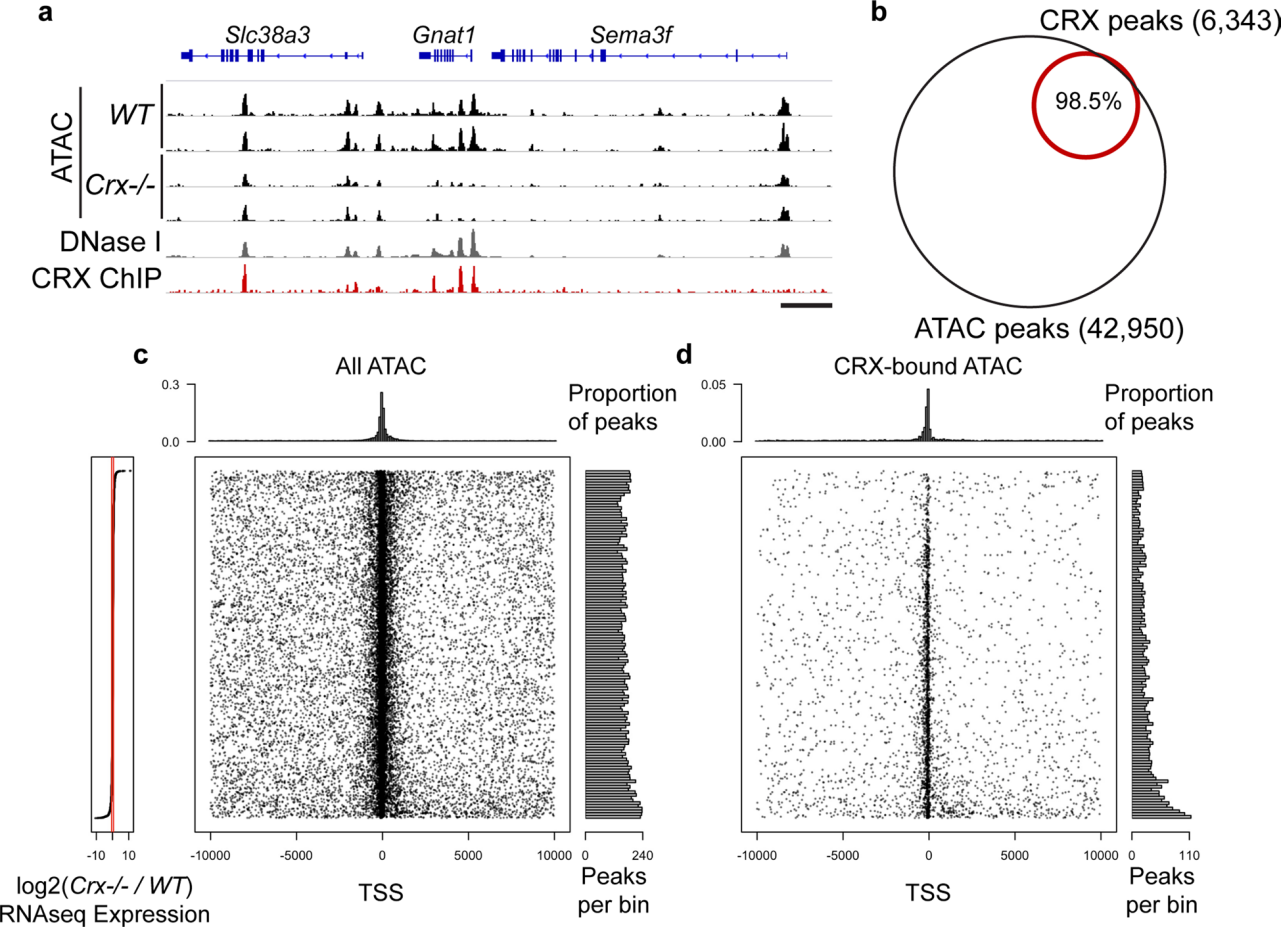
CRX binds a subset of active regulatory sites in the rod photoreceptor. **a** Browser track displays ATAC-seq, DNase I, and CRX ChIP-seq read depth. (Scale bar 5 kb) **b** Venn diagram depicting number of CRX ChIP-seq-defined binding sites that overlap with regulatory sites defined by ATAC-seq. (**c**, **d**) Meta-gene plots of all genes expressed in P21 *WT* and *Crx*−*/*− retinas, ordered by [log2] fold-change (as detailed on left). Black dots represent the center of ATAC regulatory site relative to TSS of **c** all ATAC peaks and of **d** only the subset bound by CRX. Histograms on X and Y axes display density and distribution of the data points

To determine active regulatory regions in the genome of mouse rod photoreceptors, we performed whole retina ATAC-seq [35], a technique that profiles open chromatin regions. We chose to profile at postnatal day 14 (P14), because all retinal cell types are born by this age [6] and photoreceptor specification is completed. Replicate experiments on whole retina of *C57BL/6* *J* (*WT*) mice, where rods comprise ~80% of the cells [1, 5], were highly reproducible (*r* = 0.99) and very consistent with ENCODE DNase I hypersensitivity profiles of adult (8wk) whole retina (r ≥ 0.92) (Fig. 1a and Additional file 2: Fig. S1) [29, 33].

We overlapped these regulatory sites (ATAC-seq peaks) with sites bound by CRX (detected by ChIP-seq) [21]. The majority (>98%) of CRX-bound sites were contained within ATAC-sensitive genomic regions, but many ATAC-sensitive sites showed no CRX enrichment (Fig. 1a, b).

To understand the role of these regulatory elements, we first analyzed their distribution around the transcription start site (TSS) of each gene expressed in the retina in the context of that gene’s dysregulation in *Crx*−*/*− cells. Figure 1c, d plots the TSS +/− 10 kb of each gene ordered along the y-axis by the expression change in the *Crx*−*/*− (detailed on left-most panel). Figure 1c details the center of every ATAC-sensitive regulatory site (black dots), the distribution of which is quantified by histograms along the top and right of the plot. As expected, these sites display a strong preference to be located near the TSS and there was no preference for these sites to be near genes that were differentially regulated in the *Crx*−*/*− retina. We also plotted in the same manner only the subset of ATAC regions bound by CRX (Fig. 1d). These sites showed a similar preference to be located near the TSS. However, we were surprised to find that while there was some preference for genes that lose expression in the *Crx*−*/*− retina (excess distribution near bottom of plot), many CRX-bound sites were near genes that are not transcriptionally affected upon its loss. These data suggested that while CRX is a strong transcriptional activator [10], not every binding site has the same regulatory potential or dependency upon CRX activity.

### *Crx*−*/*− retinas have an altered photoreceptor epigenome

**Fig. 2:**
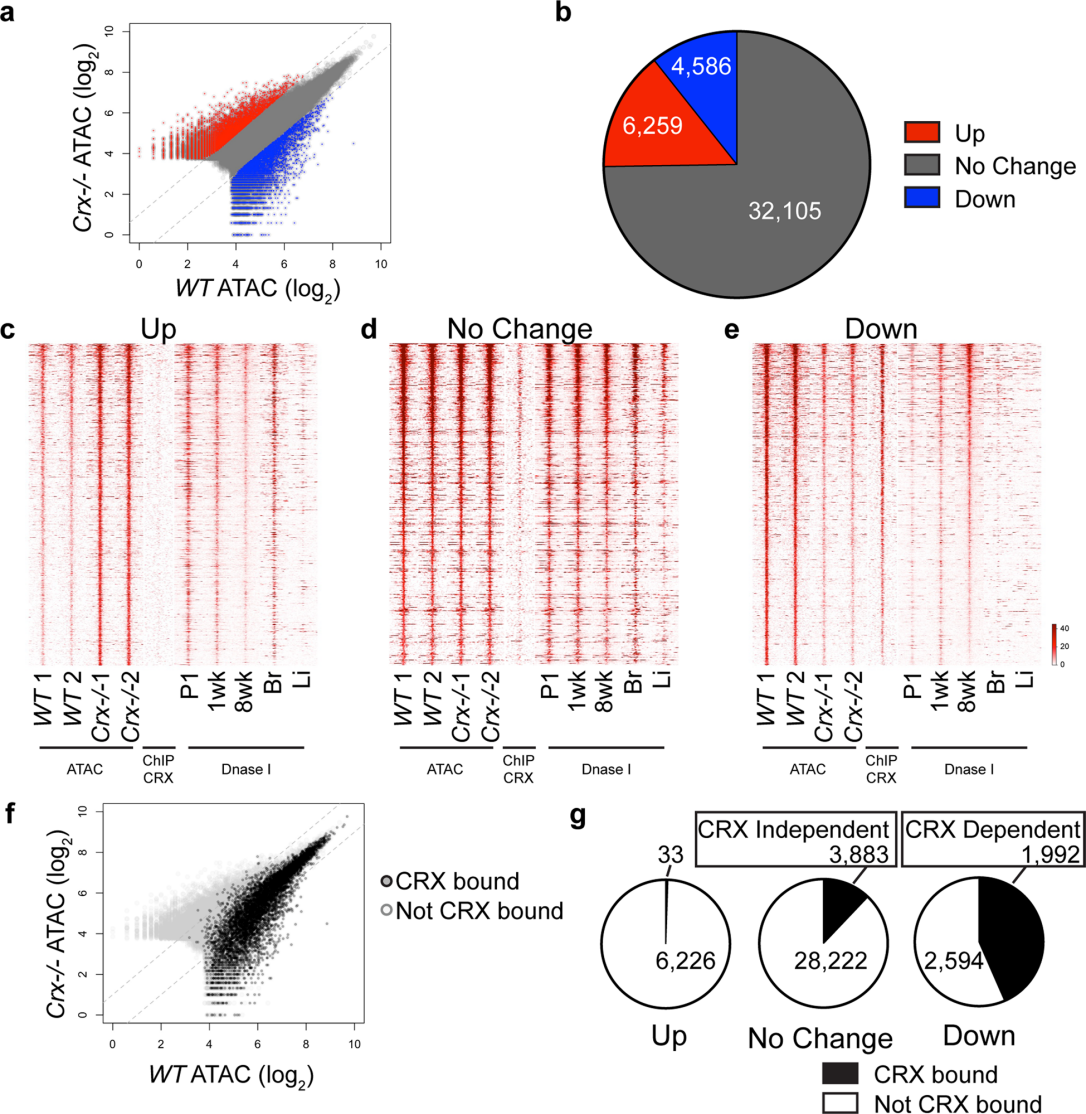
Loss of CRX affects many developmentally remodeled regulatory sites. **a**, **b** Comparison of *WT* and *Crx*−*/*− ATAC-seq data shows highly disturbed epigenome in *Crx*−*/*− retinas where significant numbers of sites display increased (red) or decreased (blue) signal. **c**, **e** Read density heatmaps display reproducibility of ATAC experiments (*WT* 1 & 2 vs. *Crx*−*/*− 1 & 2), overlap of CRX binding signal, and regulatory activity as defined by DNase I in P1, 1wk, and 8wk retina, brain, and liver samples. **f** Scatterplot displays distribution of the subset of CRX-bound ATAC sites (black dots) relative to all *WT* versus *Crx*−*/*− ATAC-sensitive sites (gray). **g** Proportion of total sites within each ATAC class bound by CRX (black) are displayed in pie charts

To determine the functional implications of the loss of CRX on the epigenome of photoreceptors, we also performed duplicate ATAC-seq experiments on P14 *Crx*−*/*− retinas and compared the results to those of *WT* retinas. Again, *Crx*−*/*− replicates were highly consistent with one another (Fig. 1a and Additional file 2: Fig. S1). Although many of the ATAC peaks qualitatively resembled the *WT* signal, some (e.g., those spanning *Gnat1*) appeared much weaker in *Crx*−*/*− than *WT* (Fig. 1a). Since no photoreceptor degeneration or cell death is detected in *Crx*−*/*− at this age [19, 22], differential ATAC signals between the two mouse lines are indicative of changes in the photoreceptor epigenome. Indeed, by quantitative comparison of *WT* and *Crx*−*/*− datasets, *Crx*−*/*− retinas display a widely disturbed epigenome with both increased (“up”) or decreased (“down”) activity at potential regulatory sites (Fig. 2a). Roughly 25% of ATAC peaks were altered, with virtually equivalent proportions “up” as those that were “down” (Fig. 2b).

### *Crx*−*/*− photoreceptors fail to close and open developmentally modulated regulatory sites

Previous DNase I hypersensitivity data analyzed three stages of retinal development: P1, P7 and Adult [29, 33]. Because of the overrepresentation of rods in the mouse retina, we can generalize these samples to represent precursor, immature, and mature rod photoreceptors, respectively. Comparison to these datasets delineated distinct patterns in each class of genomic sites (Fig. 2c–e and Additional file 3: Fig. S2). Sites that were not changed between *WT* and *Crx*−*/*− were already open at early stages (Fig. 2d and Additional file 3: Fig. S2b). However, sites that are affected display contrasting dynamics over the course of development: The set that loses activity in the *Crx*−*/*− retina normally would be activated, while the set that gains activity in the mutant retina would be closed over normal development (Fig. 2c vs. 2e and Additional file 3: Fig. S2a vs Fig. S2c).

These sites also show differential tissue specificity. When compared to other DNase I data, unaffected sites are also generally active in the brain and liver (Fig. 2d). Again, affected sites show contrasting patterns, where those that lose accessibility in *Crx*−*/*− retinas are largely retina specific (Fig. 2e), while those that gain accessibility in mutant retinas are also very active in the brain (Fig. 2c). These data suggest that *Crx*−*/*− retinas maintain a majority of their basic epigenetic state that would be similar in all cell types. However, they fail to activate highly photoreceptor-specific set of sites and to inactivate many sites that are used generally in unspecified neurons.

Next, we tested what types of genes were likely regulated by these sites (Additional file 4) [38]. “Down” sites are highly enriched for gene ontology (GO) categories involved in “phototransduction,” “detection of light,” and microtubule-related processes. “Up” sites were enriched for categories involving general development or differentiation of oligodendrocytes, somatic motor neurons, and peripheral nervous system. There was also an enrichment for a single retinal-related category “detection of visible light.” Further investigation discovered the enriched genes to primarily be cone related (*Cnga*, *Cngb*, and *Gnat2*, among others), which are present in developing “immature” rods or transfated S-cones in the absence of NRL [13, 40].

### CRX binding is strongly enriched at sites that lose activity in *Crx*−*/*−

To understand the primary role of CRX in this dynamic chromatin environment, we classified ATAC sites based on the presence of an overlapping CRX ChIP signal (Fig. 2f, g). Nearly half of the ATAC peaks that are reduced in the *Crx*−*/*− retina are bound by CRX [CRX Dependent] compared to a very small fraction (< 1%) of those ATAC peaks that show increased signal (Fig. 2f, g and 2c vs. e). There was also a significant enrichment of CRX binding within ATAC sites that were unchanged in the *Crx*−*/*− retina [CRX Independent] (Fig. 2d, f, and g; Table 1).

**Table 1:** ATAC-seq peaks lost in *Crx*−*/*− show enrichment for CRX binding in *WT*

	Total	CRX-bound	%
Up	6259	33	0.5
No change	32,105	3883	12.1
Down	4586	1992	43.4

We next sought to determine the relationship between changes in ATAC-measured accessibility and local gene expression. CRX-bound sites with decreased ATAC signal in *Crx*−*/*− compared to *WT* show a strong preference for the TSS of genes that lose expression (Fig. Additional file 5: Fig. S3f). A similar trend was also observed for non-CRX-bound sites that lose ATAC signal in *Crx*−*/*− retina (Additional file 5: Fig. S3e). Conversely, ATAC sites that show increased accessibility in the *Crx*−*/*− retina, nearly all of which lack CRX binding, are greatly enriched near genes that increase expression in the *Crx*−*/*− retina (Additional file 5: Fig. S3a and S3b). Sites unchanged in the mutant retina show no strong association with any changes in gene expression (Additional file 5: Fig. S3c and S3d). Together, these data show the strong correlation between local regulatory site activity and gene expression, but highlight differences in CRX-binding sites.

### CRX acts within a variety of chromatin environments

**Fig. 3:**
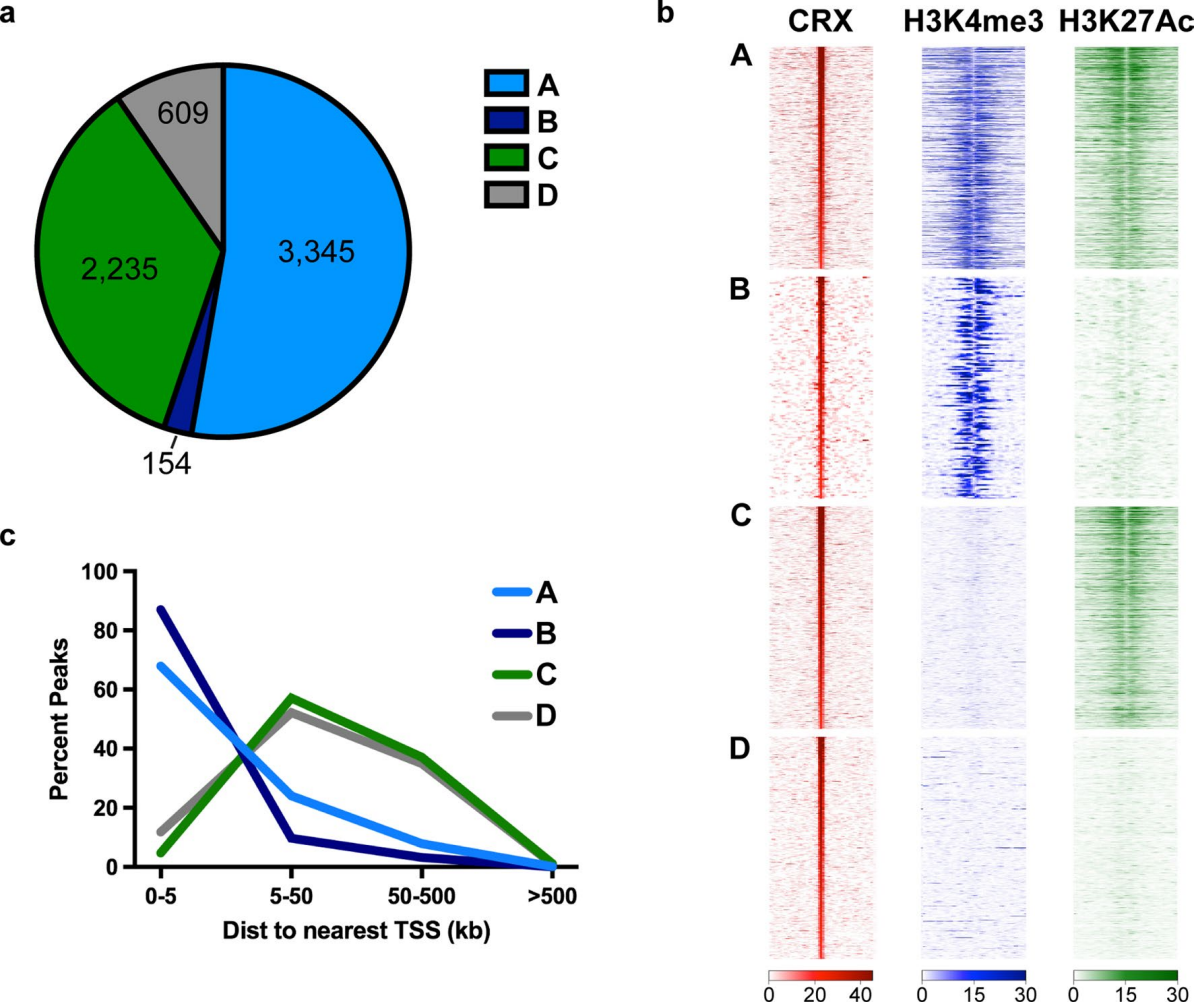
CRX binds within four chromatin environments. **a** Pie chart depicts number of CRX-binding sites that reside within Groups A–D as defined by overlap with (**b**) ChIP-seq data of H3K4me3 and H3K27Ac datasets in P14 *WT* retina. **c** Lines represent proportion of peaks within Groups A–D in each distance bin relative to nearest annotated TSS

We decided to explore the differences between these CRX-binding sites that were lost [CRX Dependent] versus maintained [CRX Independent] in the *Crx*−*/*− retina. Previous studies have classified TF binding sites into promoter or enhancer classes based on distance from the TSS. This approach is straightforward, but with new studies showing the breadth of local regulatory domains [41, 42] and the unique TSS usage of photoreceptors [43] it likely does not reflect the true biology or regulatory role of each site. Instead, we decided to distinguish binding sites based on the local epigenetic state. CRX-binding sites were located in four distinct chromatin environments defined by ChIP-seq of H3K4me3 and H3K27Ac at P14 in *WT* retinas (Fig. 3a, b). Groups A and C represent the largest proportion of the CRX-binding sites and are located in local regulatory domains and distal enhancers, respectively (Fig. 3). Group D represents CRX sites where neither active histone mark is present, and these are located similar to distal enhancer Group C, far from annotated genes (Fig. 3c). Group B includes a very small number of CRX sites that are near genes expressed at a very low level (Fig. 3c and Additional file 6: Fig. S4). We found a relatively equal representation of CRX Dependent and Independent sites within the three main Groups (Table 2), although Group A displayed some overrepresentation of Independent sites.

**Table 2:** Regulatory sites are either Dependent or Independent of CRX action

	Dependent	Independent
Group A	776	2445
Group B	3	150
Group C	998	1133
Group D	234	287

### CRX is required to activate a subset of local regulatory regions

**Fig. 4:**
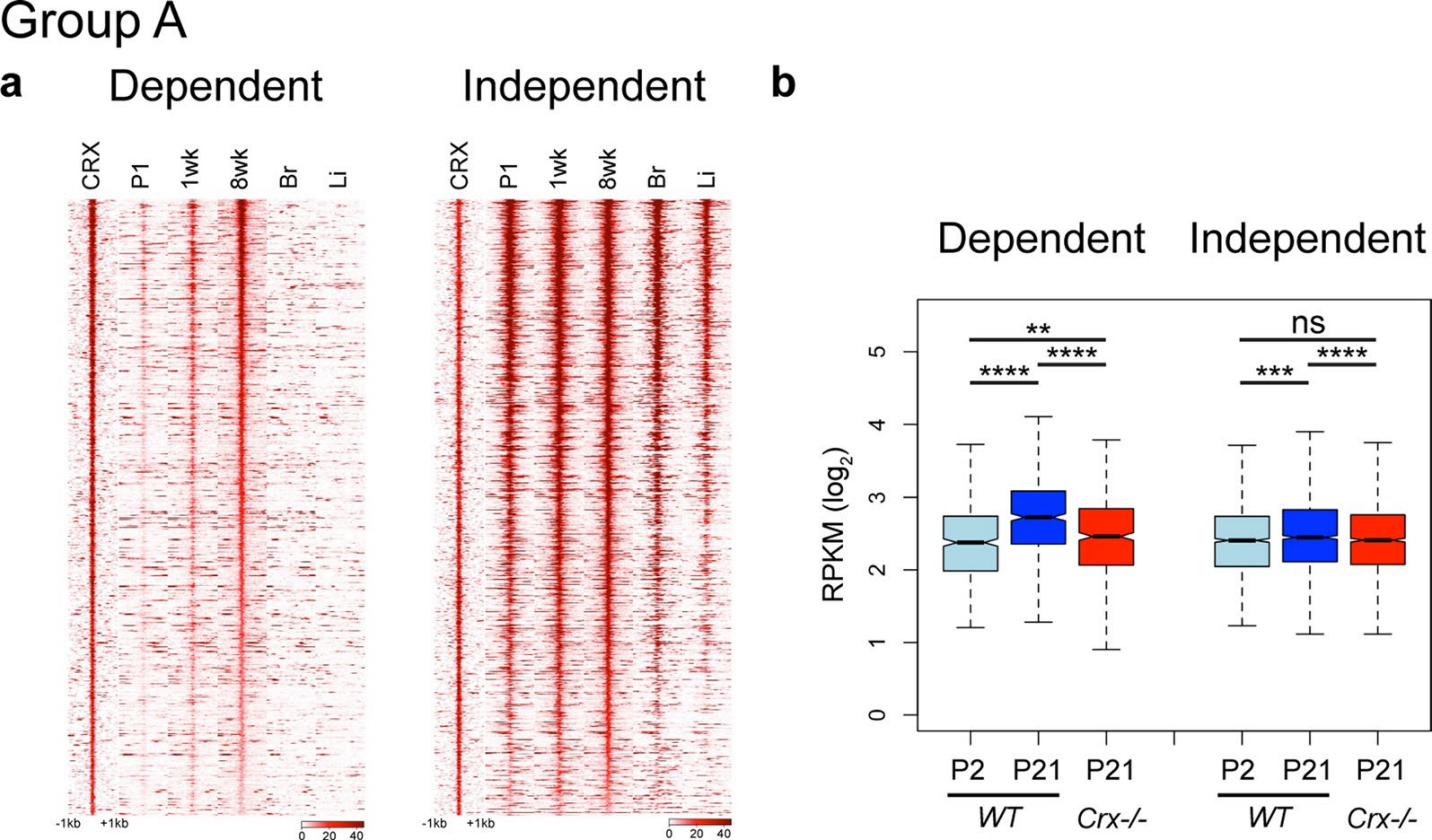
CRX is only required for activity and remodeling of a subset of Group A local regulatory sites. **a** Plots display read density of DNase I experiments centered on CRX-binding site of Dependent and Independent Group A sites. **b** Analysis of RNA-seq of nearest gene to each peak, displayed as boxplot of normalized RPKM values at P2 and P21 in *WT* and P21 in *Crx*−*/*−. (Wilcoxon rank sum test, paired; *p < 0.05, **p < 2.2 × 10^−5^, ***p < 2.2 × 10^−10^, ****p < 2.2 × 10^−16^)

To investigate the differential influence of CRX at local regulatory regions (Fig. 3; Group A), we first analyzed the temporal changes in the activation state as profiled by DNase I (Fig. 4 and Additional file 7: Fig. S5). CRX Dependent sites show strong retinal specificity and developmental activation (Fig. 4a (left panel), Additional file 7: Fig. S5a). This pattern is in stark contrast to Independent sites that display strong signal also in brain and liver samples and retinal accessibility at all ages (Fig. 4a (right panel), Additional file 7: Fig. S5b).

To further explore the epigenetic state of these sites over normal retinal development, we analyzed recently published ChromHMM data (Additional file 8: Fig. S6) [30]. By analyzing individual ChIP datasets (data not shown) and the aggregate of this data in the HMM classification of the binding sites (Additional file 8: Fig. S6a and S6b), we found that Independent sites were nearly all classified as active HMM classes throughout development, while Dependent sites displayed temporal remodeling up to P7.

To determine whether the loss of CRX affected the local histone modifications at Dependent sites, we performed ChIP-seq for H3K4me3 in *WT* and *Crx*−*/*− retinas. Peaks containing CRX Independent sites largely maintained H3K4me3 presence in mutant retinas, while peaks containing CRX Dependent sites display a significant loss of signal in *Crx*−*/*− (Fig. Additional file 7: Fig. S5c and S5d).

We sought to determine the effects of these epigenetic changes on gene expression. RNA-seq data profiling *WT* development and changes in *Crx*−*/*− were consistent with ATAC and DNase I data. Genes near CRX Independent sites show few developmental expression changes and are relatively unaffected in mutant retinas. In contrast, those near Dependent sites increase dramatically over development but largely fail to do so in the *Crx*−*/*− (Fig. 4b). Gene ontology (GO) analysis of these genes suggests Dependent genes are largely photoreceptor related, while the Independent set comprises some genes with photoreceptor function, but also genes with more general functions (protein transport, RNA processing, protein localization, etc; Additional file 4).

### CRX is required to activate a set of distal enhancers

**Fig. 5:**
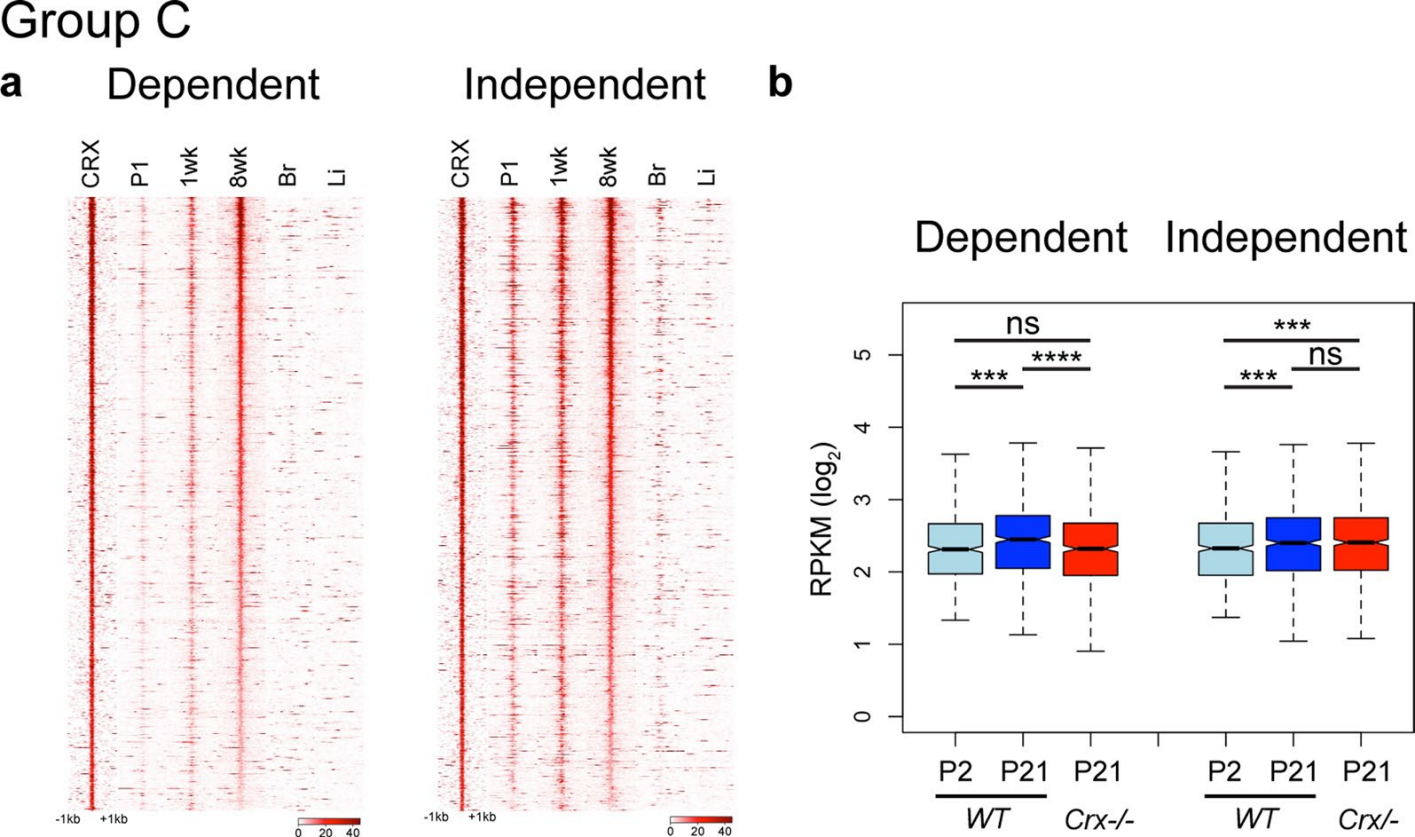
CRX activates a subset of Group C distal enhancers over development. **a** Plots display read density of DNase I experiments centered on CRX-binding sites of Dependent and Independent Group C sites. **b** Analysis of RNA-seq of nearest gene to each peak, displayed as boxplot of normalized RPKM values at P2 and P21 in *WT* and P21 in *Crx*−*/*−. (Wilcoxon rank sum test, paired; *p < 0.05, **p <2.2 × 10^−5^, ***p < 2.2 × 10^−10^, ****p < 2.2 × 10^−16^)

CRX also binds sites marked uniquely by H3K27Ac (Fig. 3; Group C), traditionally thought to be active distal enhancers. Dependent and Independent sets of enhancers both displayed dynamic changes over development and a high degree of retinal specificity (Fig. 5a and Additional file 9: Fig. S7a and S7b). However, by closer comparison of the temporal dynamics of this activation, we observed that Independent sites showed earlier activation than Dependent sites (Additional file 9: Fig. S7a and S7b). HMM classification was nearly identical by P14, but comparison of P0 and P3 data showed Independent sites switch to active classifications earlier than Dependent (Additional file 8: Fig. S6c and S6d).

To test whether ATAC signal changes correlated with the mutant epigenome, we performed ChIP-seq for H3K27Ac at P14 in *WT* and *Crx*−*/*− retinas. As expected, Dependent sites displayed a significant decrease in H3K27Ac deposition in *Crx*−*/*− retinas, while Independent sites largely maintained this mark (Additional file 9: Fig. S7c and Additional file 9: Fig. S7d).

Expression of the nearest gene to each enhancer also highlights differences between CRX-binding sites. Genes near both Dependent and Independent sites increased in expression over development in *WT* retinas (Fig. 5b). However, this increase is attenuated upon loss of CRX only in the Dependent set (Fig. 5b). GO analysis showed a very clear distinction where Dependent genes are photoreceptor related, while Independent genes have a variety of general functions (Additional file 4).

### CRX controls distal regulatory sites in the absence of active histone marks

A significant number of CRX-bound sites were not marked by either H3K4me3 or H3K27Ac (Fig. 3; Group D). When we analyzed the DNase I and HMM data at these sites, the pattern looked similar to Group C enhancers where CRX Dependent sites opened later than Independent and were more retina specific (Additional file 8: Fig. S6e, f; Additional file 10: Fig. S8a, d, e). One notable difference was that HMM classification highlighted a subset of Independent sites classified throughout development as Class 11 [Insulator] (Additional file 8: Fig. S6e).

Interestingly, gene expression data highlighted a different scenario than other groups. The expression of the nearest gene to CRX Dependent sites displayed no significant differences (Additional file 10: Fig. S8b). While differences in gene expression were statistically significant in the Independent set, the degree of change was very modest.

### Base composition and conservation differentiate CRX sites

**Fig. 6:**
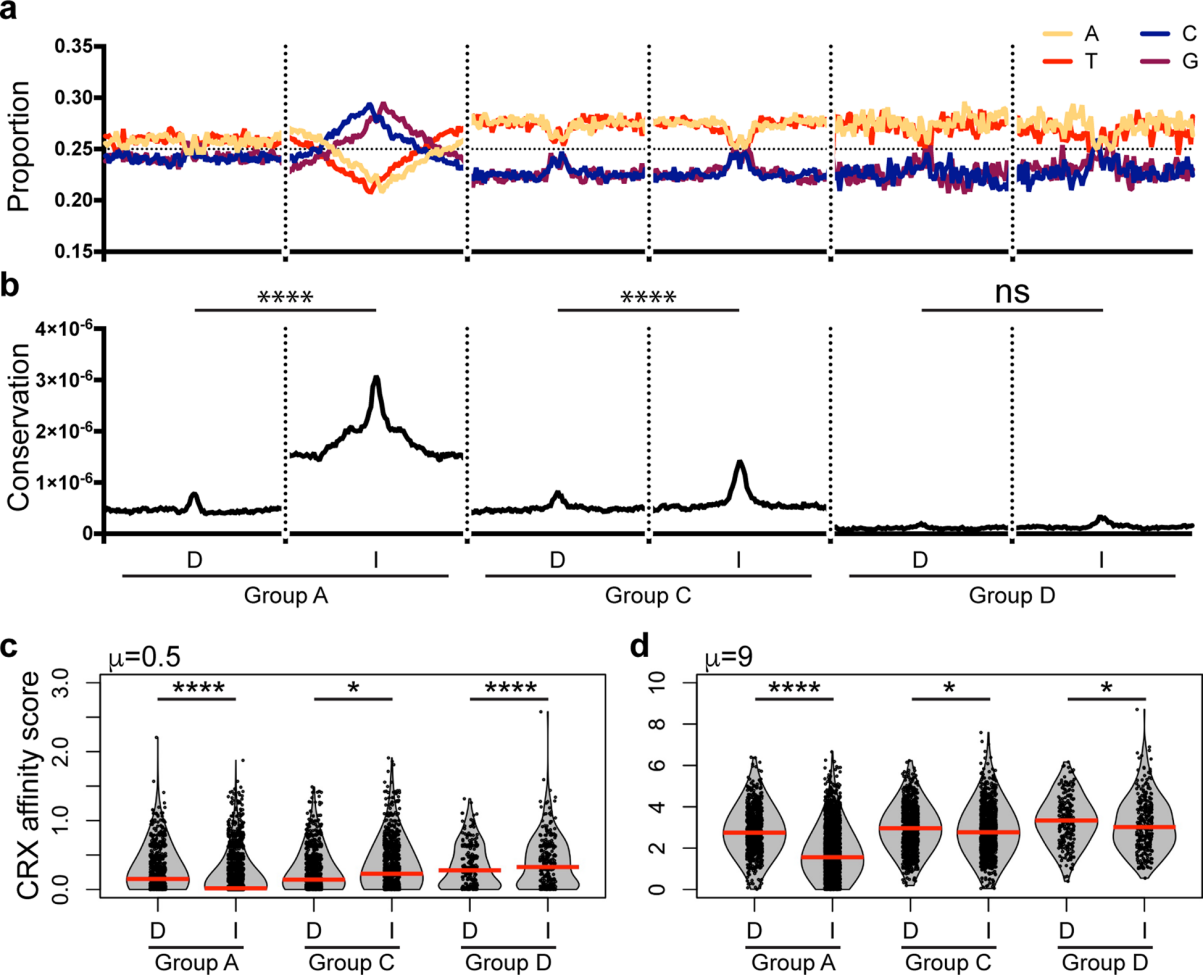
Sequence analyses differentiate Dependent and Independent sites. **a** Nucleotide density and **b** conservation scores display differences between Groups A, C, and D and between Dependent and Independent classes of CRX sites in each Group. Both are calculated in 20-bp windows +/− 1 kb from center of CRX peak. (Two-way ANOVA with Tukey multiple comparison testing; ****p < 0.0001) **c, d** CRX affinity scores, as defined by BEEML algorithm are displayed for two DNA/protein ratios (μ values). (Red line denotes median; Wilcoxon rank sum test, *p < 0.05, **p < 2.2 × 10^−5^, ***p < 2.2 × 10^−10^, ****p < 2.2 × 10^−16^)

Our epigenome analyses identified distinct types of CRX sites, but raised the question of whether sequence characteristics “code” for these differences. As base composition, such as CpG density, can determine the general regulatory role of genomic locations, we analyzed nucleotide frequency across CRX regulatory sites (Fig. 6a). Group A Dependent sites show a fairly equal nucleotide distribution, especially within the central 200 bp of the CRX-bound regulatory site. This is in dramatic contrast to the Independent Group A sites that are within highly GC rich regions. Group C and D distal regulatory elements did not show obvious differences between Independent or Dependent sites. Both have balanced content within the regulatory site, but lie within AT-rich domains.

We also analyzed the extent to which the sequences were conserved across vertebrate evolution (Fig. 6b). All groups display a central region that is more conserved than the surrounding environment, supporting the importance of the ATAC- and CRX ChIP-specified regulatory sites. However, Independent sites in Groups A and C showed significantly higher conservation than their Dependent counterparts.

### CRX has different affinity for Dependent versus Independent sites

We next sought to determine whether the predicted binding affinity of CRX could distinguish sites. We first noted that all CRX-bound sites were strongly enriched for the presence of a canonical CRX HD site compared to all ATAC-sensitive sites (Additional file 11: Fig. S9a). However, there were also significant differences that distinguished Dependent versus Independent sites. In Group A proximal regulatory regions, significantly more Dependent than Independent sites contained the canonical CRX site (Additional file 11: Fig. S9a). This difference was reflected in the CRX ChIP-seq, where Dependent sites show a higher read depth (Additional file 11: Fig. S9b). Group C and D sites displayed the opposite characteristics, where Independent sites were more likely to contain the CRX motif (Additional file 11: Fig. S9a), although there was no clear difference in the intensity of the original ChIP data (Additional file 11: Fig. S9c and S9d).

We also predicted CRX affinity using an algorithm that takes into account the relative ratio of protein to DNA [28, 44]. At both low and high ratios (*μ* values), Group A Dependent sites displayed higher binding affinity than Independent sites (Fig. 6c, d). However, when Groups C and D were analyzed at a low protein to DNA ratio (*μ *=0.5), Independent sites have a significantly higher affinity score, consistent with the traditional motif search data (Fig. 6c). When analyzed at a higher ratio, this relationship inverted and Dependent sites instead showed a higher predicted affinity (Fig. 6d). This suggests that Dependent distal regulatory sites have more low-affinity CRX-binding sites that could additively contribute to CRX recruitment or direct functional differences.

### Other TFs may compensate or be more influential at Independent sites

We also wondered if binding sites for other TFs could differentiate CRX Dependent versus Independent sites. We analyzed ± 100 bp from the center of each CRX peak for enrichment of sequences *de novo* (Additional file 12) and motifs in the JASPAR database (Additional file 13: Fig. S10a). Both methods reported the highest enriched motif in all groups to be a canonical homeobox (Additional file 13: Fig. S10a [marked by *] and Additional file 12).

The most striking difference we noted by both methods was a unique enrichment of many TF motifs in Group A Independent sites. Known TFs included KLF4, ELK1/4, E2F1, and NFYa (Additional file 13: Fig. S10a [marked by **]), while *de novo* motifs included promoter elements (GC-box), NFY, NRF1, BHLH, among others (Additional file 12).

The other five sets shared enrichment of many known factors including GATA, LHX, and NKX family motifs (Additional file 13: Fig. S10a), although there were also notable differences. Both methods suggested an enrichment of CTCF specifically in Group D Independent sites (Additional file 13: Fig. S10a [marked by ***] and Additional file 12), and known motif analysis suggested that ESRRB and FOXC1 are enriched only in Dependent sites (Additional file 13: Fig. S10a [marked by ****]). *De novo* methods also indicated strong unique enrichment of NEUROD1, MEF2D, and MEIS1 motifs in Group C Independent sites (Additional file 12).

To confirm and explore the relevance of the motif analysis, we chose to focus on the enrichment of MEF2D and CTCF sites. The CTCF motif was enriched only in Group D Independent sites by both methods. As previously discussed, this set contains many sites with Insulator function (Additional file 8: Fig. S6e [HMM Class 11 purple]). We reanalyzed CTCF ChIP-seq data and confirmed that CTCF binds at Group D Independent sites throughout development, and this binding was reasonably specific with minimal enrichment at Independent Group A sites but none at any Dependent sites (Additional file 13: Fig. S10c and S10d).

We also explored the enrichment of the MEF2D motif in Group C Independent sites (Additional file 12). Analysis of previously published ChIP-seq data [16] showed that MEF2D binds both Dependent and Independent enhancers in *WT* retina, but this signal is dramatically reduced only at Dependent sites in the *Crx*−*/*− retina (Additional file 13: Fig. S10b). Together, these data support the relevance and interpretation of the motif enrichment analyses.

## Discussion

### Activity defines two types of CRX-binding sites

By comparing the genome-wide activity changes in *WT* versus *Crx*−*/*− retinas, we have determined that the loss of CRX causes significant changes in activity at ~ 25% of all regulatory sites. Even though CRX is an activating TF, sites that increase were as prevalent as those that lose activity (Fig. 2). By analyzing these sites alongside time course DNase I hypersensitivity data, we have confirmed previously hypothesized mechanisms of *CRX*-associated disease, that the rod photoreceptors are stuck in a pre-developmental state; sites that were supposed to close remain active, and sites that were supposed to progressively open did not do so (Fig. 2e–g and Additional file 3: Fig. S2). This model of *CRX* disease explains previous findings that *Crx* mutants aberrantly express “cone”-related transcripts [7]. Together with this data, recent reports that suggest a more common developmental path of rods and cones than previously recognized [45] support the model that *Crx* mutant retinas resemble a premature state prior to the dramatic epigenetic switch toward rod photoreceptor identity.

Many of the peaks that lose activity are normally bound by CRX itself (Fig. 2f, g), consistent with these sites being Dependent on its activity. CRX showed virtually no binding at peaks with increased activity, but surprisingly did bind many peaks that were unchanged, or Independent (Fig. 2c, d). Our method that incorporates other aspects of the local epigenetic neighborhood, including two active histones, clearly establishes distinct types of CRX-binding sites beyond simply TSS proximal and TSS distal. We propose this classification system is more informative and biologically relevant.

CRX is activated in early born photoreceptors and maintains expression in mature cells. Both developmental DNase I and HMM classifications showed that all CRX Dependent sites normally undergo dramatic activation over retinal development. By ChIP-seq, we showed that H3K4me3 and H3K27Ac marks were lost at Group A and C Dependent sites, respectively, and by RNA-seq, that nearby genes were not properly activated over development in *Crx*−*/*− retina. Together, this suggests that CRX is involved in this activation likely through its direct binding and recruitment of other general TFs and co-regulators. Previous work has already shown clear roles of such co-factors including CBP/P300, Ataxin7-STAGA, DNMT1 in retinal development [17, 18, 46, 47].

### Sequence features distinguish CRX Dependent sites

**Fig. 7:**
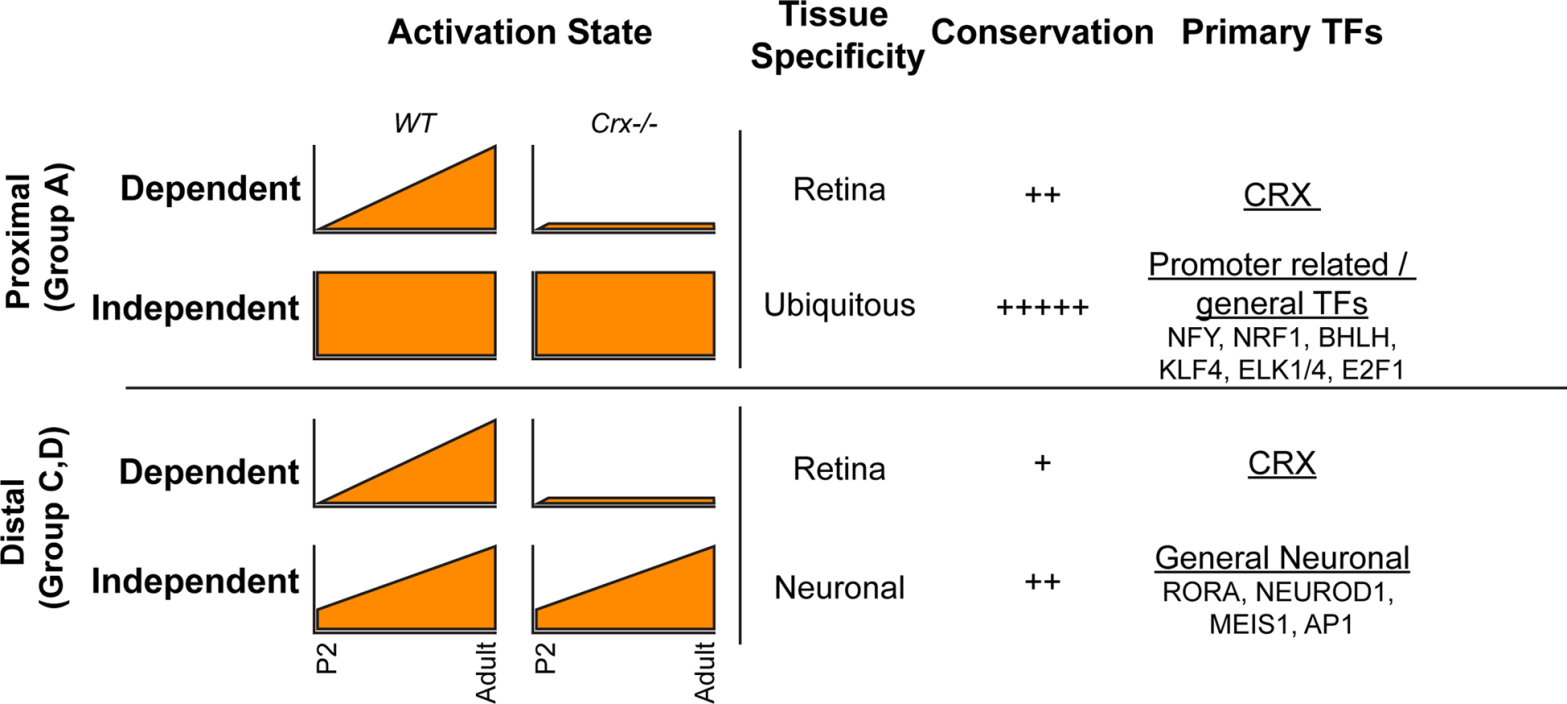
Model synthesizes new insights into difference in CRX activity and mechanism of action at CRX Dependent versus Independent proximal and distal regulatory sites. The model describes differences in activity state over time (left), and distils motif analysis and base conservation data

Independent sites showed two very different patterns (Fig. 7). Group A (TSS proximal) Independent sites were open constitutively. These regions showed no evidence of developmental remodeling and were also active in other tissues. This suggests that these sites are generally used regulatory elements, and GO analysis of the nearby genes supports this interpretation as these genes were categorized as having very general cellular functions. Conversely, Independent Group C and D sites display temporal activation and retinal specificity, but quantitative analysis of the DNase I and HMM data suggests they begin remodeling prior to CRX expression and their respective Dependent Group sites. The ability of the photoreceptor to maintain or continue activating these Independent sites and properly express nearby genes suggests that the binding of CRX here is not essential. However, we cannot formally exclude the possibility that other homeodomain TFs (such as OTX2) compensate in the *Crx*−*/*− retina.

We analyzed the affinity of CRX by two distinct methods. Both predict that CRX binds more efficiently to Dependent than Independent Group C sites, which was supported by ChIP-seq data (Additional file 11: Fig. S9). We were surprised to find that Dependent distal elements (Groups C and D) display fewer high but more low-quality motifs. Differences in activity relevant to binding affinity have been predicted for CRX [28]. This difference could establish a fundamental mechanism for CRX activity at enhancers, but alternative biochemical experiments would be necessary to study binding efficiency, on/off rates, and functional effects of these different types of sequences.

*De novo* motif analysis did not reveal any differences in the HD motif between Dependent and Independent sites. This is not unexpected, as this motif is shared among many HD TFs expressed throughout the body, including others expressed in the retina (OTX2 in particular). These results together suggest that CRX binding is directed by the presence of a strong consensus HD motif, but this association cannot be blindly interpreted as an important functional interaction (Fig. 7). At Independent regulatory sites, CRX binding may simply be the by-product of accessibility of a HD element bound by another HD TF either in the retina or important in other cell types. Only the precise mutation of these elements in the genomic context can answer this question.

Previous work had noted that CRX bound within many GC-rich genomic regions [21]. Our analysis discovered that these constitute proximal Independent regulatory sites. While GC-rich areas are normally thought to represent repressed CpG islands, other reports have noted that ubiquitously expressed promoters display high GC content while cell-type-specific promoters are AT rich [48]. We expected that CRX Dependent sites would be very highly conserved across species. While it was the case that the regulatory elements were more conserved than flanking sequences, Independent sites were more conserved than Dependent counterparts. *De novo* and known motif analyses discovered that Independent sites, especially Group A promoters, were enriched for a variety of TF motifs. Together, these data suggest that Independent sites are activated in other cell types of the body and are regulated by shared or more general TFs.

Analysis of Dependent sites did suggest several CRX interacting partners, although motifs for known partners NRL and NR2E3 that synergize or bind with CRX [12, 49] were not observed. This supports the model that while all three factors are necessary for proper gene expression, CRX acts as the primary sequence specific targeting factor for the three as a complex. This is supported by analysis showing that ChIP-seq of NRL largely recapitulates that of CRX (Ruzycki and Chen, unpublished results). Group C sites in particular displayed enrichment for a number of other factors including NKX, ESRRB, and FOXC. These could represent new TFs that either act in coordination with CRX to activate these enhancers or could represent pioneer factors that opened the sites to allow for CRX to bind and fully activate.

Our analyses have emphasized that not every TF binding site is equal. While CRX or any other homeodomain TF may bind thousands of sites, only a subset of those sites (for CRX, < 1/3) require the TF to establish or maintain the local epigenetic state. We propose these elements and the homeodomain motifs within Dependent sites are excellent candidates where non-coding variants may cause human retinal disease. Our findings are based on an embryonic loss-of-function study. In this setting, CRX acts as an “acceleratory factor” required for enhancing Dependent site activity. However, these experiments do not address whether CRX is sufficient for achieving the active chromatin state and when CRX’s epigenomic activity is required. Ectopic expression of CRX in cultured HEK293 and Y79 retinoblastoma cells failed to produce a rod-like epigenome, even with co-expression of NRL (Ruzycki and Chen, unpublished results), suggesting CRX is not a “pioneer” factor that can bind to fully closed sites to induce *de novo* chromatin remodeling for cell fate specification. Instead, CRX appears only able to act on sites that have been “primed” for photoreceptor rearrangement in the precursor cells. Indeed, even CRX Dependent sites show some level of ATAC sensitivity, perhaps evidence for prior opening by another factor, although we cannot exclude that this is the result of some compensation by another TF in the absence of CRX. Future studies, such as temporal knockout or ectopic expression of CRX in developing or mature photoreceptors, are needed to address the sufficiency and plasticity questions, important for understanding and treating CRX-linked diseases.

## Conclusions

Our in-depth analysis of the epigenomic function of CRX highlights the complex nature of TF-mediated gene regulation. Our data specify the subset (~ 1/3) of sites bound by CRX *in vivo* that require CRX for proper epigenomic activation. While our study focuses on the role of a specific TF, CRX, during retinal development, our findings uncover novel principles that are likely applicable to TFs in other tissue systems. Our methods also illustrate the utility of aggregating newly generated data with publicly available datasets as a powerful way to reveal potential mechanisms for TF activity and pinpoint candidates for further study.

## Additional files

**Additional file 1**. Analysis and quantification of ATAC-seq and ChIP-seq data. Excel sheets report; ATAC-seq peaks and genotypic comparison, CRX ChIP binding sites and classification into Groups A–D, and histone ChIP-seq quantification.

**Additional file 2: Fig. S1**. ATAC-seq data show strong correlation between replicates and with Dnase I hypersensitivity data. Scatterplots display normalized read counts (CPM) within ATAC-seq determined regulatory sites, and values represent pairwise Pearson correlation coefficient

**Additional file 3: Fig. S2**. Affected regulatory sites show distinct changes in activity over normal retinal development. Plots depict average (black line, gray bars +/- SEM) of Dnase I hypersensitivity data presented in Fig. 2c–e for three ages indicated of sites increased (**a**) not changed (**b**) or decreased (**c**) in the *Crx−/−* retina compared to *WT*. (Two-way ANOVA with Tukey multiple comparison testing; ****p <0.0001

**Additional file 4.** GO analysis describes differences in nearest gene to each regulatory site. Sheets within the table represent distinct comparisons referenced within the text.

**Additional file 5: Fig. S3**. CRX-bound ATAC peak signal changes correlate with expression changes of nearby genes. (**a, c, e—left panel**) Meta-gene plots of all genes expressed in P21 *WT* and *Crx-/-* retinas, ordered by [log2] fold-change (as depicted in plot on left). Black dots represent the center of ATAC regulatory site relative to TSS of all ATAC peaks not bound by CRX (**a**, **c**, **e—right panel**) and of only the subset bound by CRX (**b**, **d**, **f**). Peaks are divided by their changes in ATAC signal sites increased in *Crx-/-* relative to *WT* (**a**, **b**), those that are not changed (**c**, **d**), and those that decrease in *Crx−/−* (**e**, **f**). Histograms of X and Y axes display density and distribution of the d.

**Additional file 6: Fig. S4**. Distinct group-related genes show different expression values. Boxplots represent normalized expression (RPKM) of the nearest gene to each peak within Groups A–D.

**Additional file 7: Fig. S5**. CRX is required to activate Dependent Group A local regulatory elements and to remodel chromatin. Plots display mean (black line) and SEM (gray bars) of DNase I data presented in Fig. 4a for CRX Dependent (**a**) and Independent (**b**) sites. (Two-way ANOVA with Tukey multiple comparison testing; ****p <0.0001). **c** Scatterplot displays changes in H3K4me3 deposition at all peaks (light gray). Black and red denote the subset of H3K4me3 peaks that contain Independent and Dependent Group A sites. **d** Quantification of the fold-change of H3K4me3 deposition in *WT* versus *Crx−/−*. (Wilcoxon Rank Sum Test; ****p < 2.2 × 10^−16^).

**Additional file 8: Fig. S6**. Dependent and Independent sites show different chromatin state dynamics. **(a, c, e**) Stacked bargraphs represent proportion of Group A–D sites that are contained within each HMM-defined chromatin state at 8 developmental ages. (**b, d, f**) Quantification of data binned into HMM classes 1–4, 5–7, 8–10, and 11, for Dependent and Independent sites show different dynamics of reorganization over development. Legend defines basic classification of HMM classes [30].

**Additional file 9: Fig. S7**. CRX is required to activate Dependent Group C enhancer elements and remodel chromatin. Plots display mean (black line) and SEM (gray bars) of DNase I data presented in Fig. 5a for CRX Dependent (**a**) and Independent (**b**) sites. (Two-way ANOVA with Tukey multiple comparison testing; **** p<0.0001) (**c**) Scatterplot displays changes in H3K27Ac deposition at all peaks (light gray). Black and red denote the subset of H3K27Ac peaks that contain CRX Independent and Dependent Group C sites. (**d**) Quantification of the fold-change of H3K27Ac deposition in *WT* vs *Crx-/-*. (Wilcoxon Rank Sum Test; **** p < 2.2 × 10^−16^)

**Additional file 10: Fig. S8**. CRX is required to activate Dependent Group D distal regulatory elements. **a** Plots display read density of DNase I experiments centered on CRX-binding site of Dependent and Independent Group D sites. **b** Analysis of RNA-seq of nearest gene to each peak, displayed as boxplot of normalized RPKM values at P2 and P21 in *WT* and P21 in *Crx-/-*. (Wilcoxon Rank Sum Test, Paired; *p < 0.05, **p < 2.2 × 10^−5^, ***p < 2.2 × 10^−10^, ****p < 2.2 × 10^−16^) Plots display mean (black line) and SEM (gray bars) of DNase I data above for CRX Dependent (**c**) and Independent (**d**) sites. (Two-way ANOVA with Tukey multiple comparison testing; ****p < 0.0001).

**Additional file 11: Fig. S9**. Sites display different presence of CRX motif and binding. **a** Proportion of sites within each of the noted groups that contains the published CRX motif. (Fisher’s exact test; ***p = 0.0001, ****p < 0.0001) **b–d** Quantification of read depth of CRX ChIP-seq at Dependent (red) and Independent (blue) sites within the specified Groups.

**Additional file 12.** De novo motif analysis identifies sequences enriched in different sets of CRX-bound regions. Data presented in columns A–C & E–I are generated by HOMER (see “Methods”). Column D represents the scanning of the identified sequence back across the 6 classes of CRX sites (+/− 1kb from peak center). The plot in line 1 depicts the layout of the plot, and the *y* axis quantifies ‘motifs per bp.’

**Additional file 13: Fig. S10**. TF motifs explain nature of Independent site activation in absence of CRX. **a** Heatmap shows unsupervised clustering of [−log10] transformed p-values representing the significance of representation of the noted TF motif within the set of sites. (* -****) represent TFs referenced in details in the text. **b** Quantification of MEF2D ChIP-seq data at Group C enhancer sites displays loss of signal specifically at Dependent sites (red) in the *Crx−/−*. Independent sites (blue) show no change in signal. Quantifications of CTCF binding over time (**c** maximum peak intensity over development and **d** relative to CRX bindings site at P21) display consistent signal at Group D Independent sites.

## Abbreviations

CRX: cone rod homeobox protein
NRL: neural retina-specific leucine zipper protein
ATAC-seq: assay for transposase-accessible chromatin using sequencing
ChIP-seq: chromatin immunoprecipitation followed with deep sequencing
H3K4me3: histone three lysine four trimethylation
H3K27Ac: histone three lysine twenty-seven acetylation
