# Supplementary figures and images for: CRX directs photoreceptor differentiation by accelerating chromatin remodeling at specific target sites
Source: Epigenetics Chromatin. 2018 Aug 1;11:42. doi: 10.1186/s13072-018-0212-2 (PMC6069558; doi:10.1186/s13072-018-0212-2)

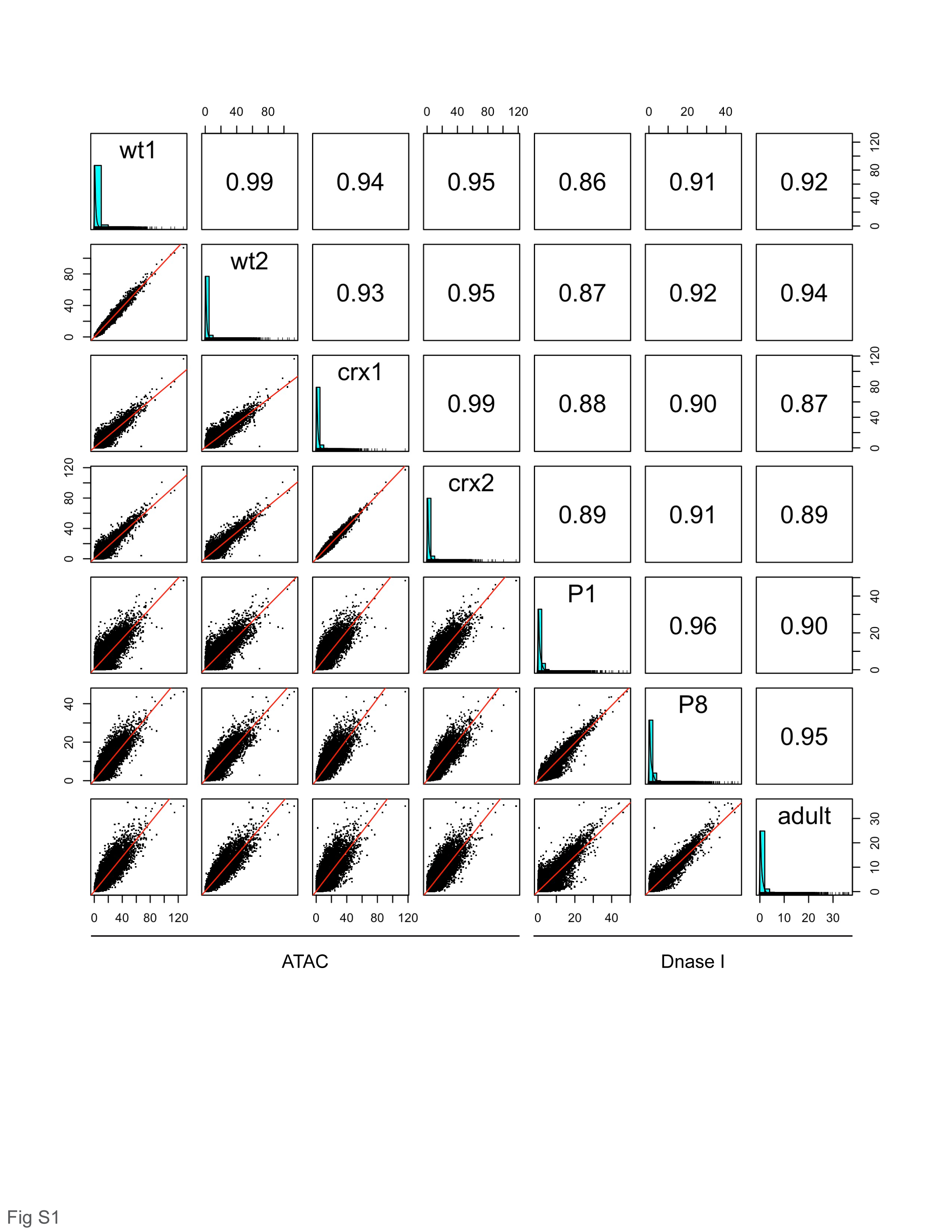

Supplement: Supplementary file 2 — Additional file 2: Fig. S1. ATAC-seq data show strong correlation between replicates and with Dnase I hypersensitivity data. Scatterplots display normalized read counts (CPM) within ATAC-seq determined regulatory sites, and values represent pairwise Pearson correlation coefficient [file 13072_2018_212_MOESM2_ESM.tif]

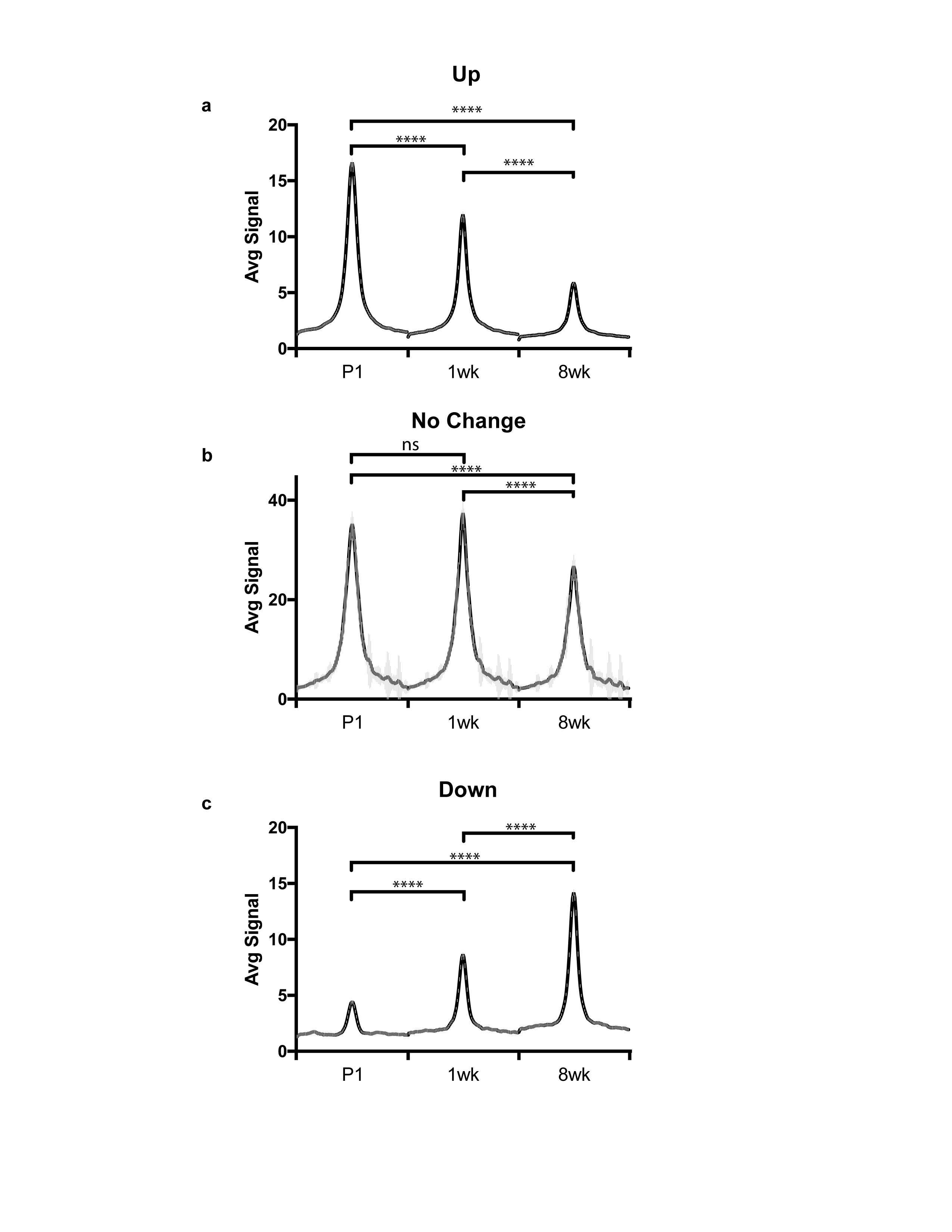

Supplement: Supplementary file 3 — Additional file 3: Fig. S2. Affected regulatory sites show distinct changes in activity over normal retinal development. Plots depict average (black line, gray bars +/- SEM) of Dnase I hypersensitivity data presented in Fig. 2c–e for three ages indicated of sites increased (a) not changed (b) or decreased (c) in the Crx−/− retina compared to WT. (Two-way ANOVA with Tukey multiple comparison testing; ****p <0.0001 [file 13072_2018_212_MOESM3_ESM.tif]

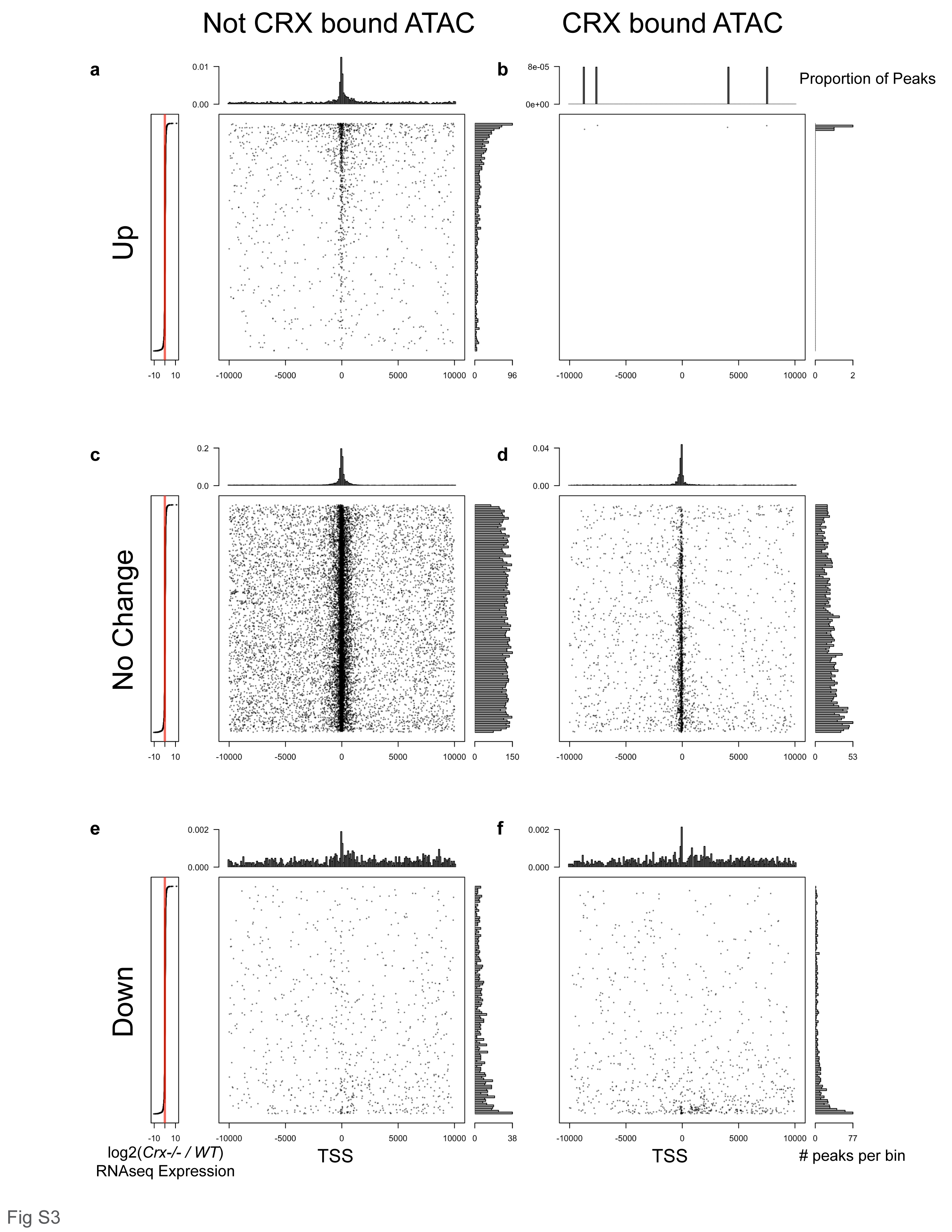

Supplement: Supplementary file 5 — Additional file 5: Fig. S3. CRX-bound ATAC peak signal changes correlate with expression changes of nearby genes. (a, c, e—left panel) Meta-gene plots of all genes expressed in P21 WT and Crx-/- retinas, ordered by [log2] fold-change (as depicted in plot on left). Black dots represent the center of ATAC regulatory site relative to TSS of all ATAC peaks not bound by CRX (a, c, e—right panel) and of only the subset bound by CRX (b, d, f). Peaks are divided by their changes in ATAC signal sites increased in Crx-/- relative to WT (a, b), those that are not changed (c, d), and those that decrease in Crx−/− (e, f). Histograms of X and Y axes display density and distribution of the d. [file 13072_2018_212_MOESM5_ESM.tif]

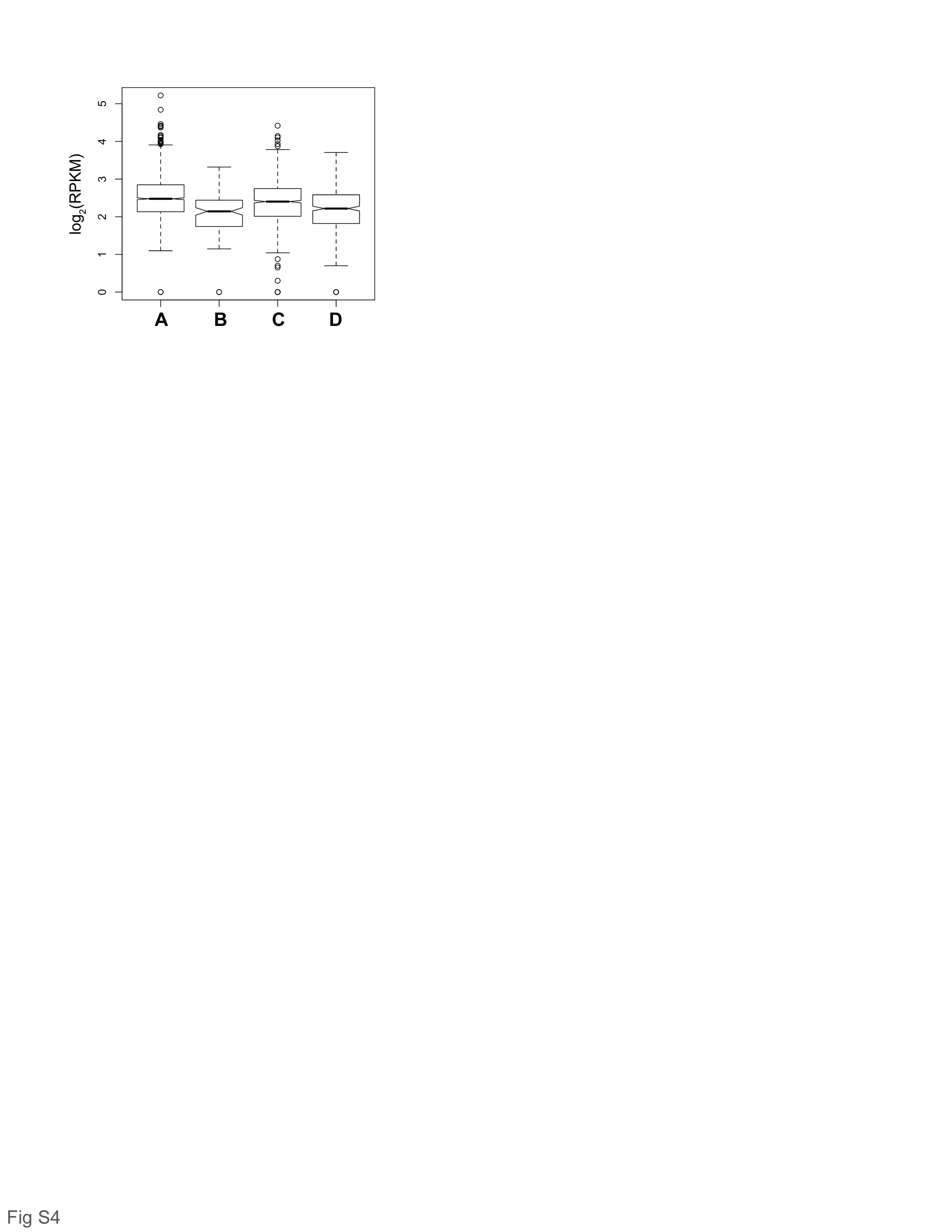

Supplement: Supplementary file 6 — Additional file 6: Fig. S4. Distinct group-related genes show different expression values. Boxplots represent normalized expression (RPKM) of the nearest gene to each peak within Groups A–D. [file 13072_2018_212_MOESM6_ESM.tif]

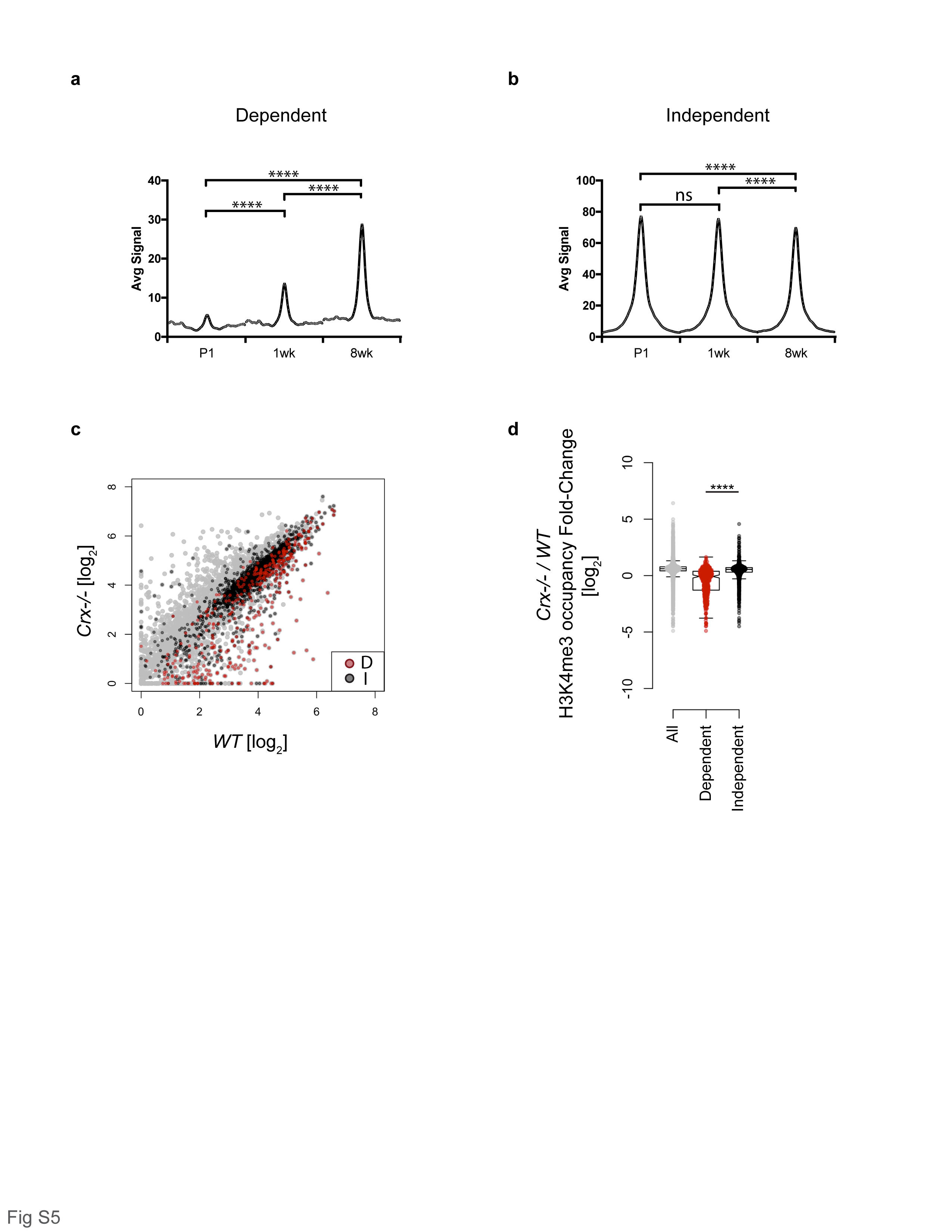

Supplement: Supplementary file 7 — Additional file 7: Fig. S5. CRX is required to activate Dependent Group A local regulatory elements and to remodel chromatin. Plots display mean (black line) and SEM (gray bars) of DNase I data presented in Fig. 4a for CRX Dependent (a) and Independent (b) sites. (Two-way ANOVA with Tukey multiple comparison testing; ****p <0.0001). c Scatterplot displays changes in H3K4me3 deposition at all peaks (light gray). Black and red denote the subset of H3K4me3 peaks that contain Independent and Dependent Group A sites. d Quantification of the fold-change of H3K4me3 deposition in WT versus Crx−/−. (Wilcoxon Rank Sum Test; ****p < 2.2 × 10^−16^). [file 13072_2018_212_MOESM7_ESM.tif]

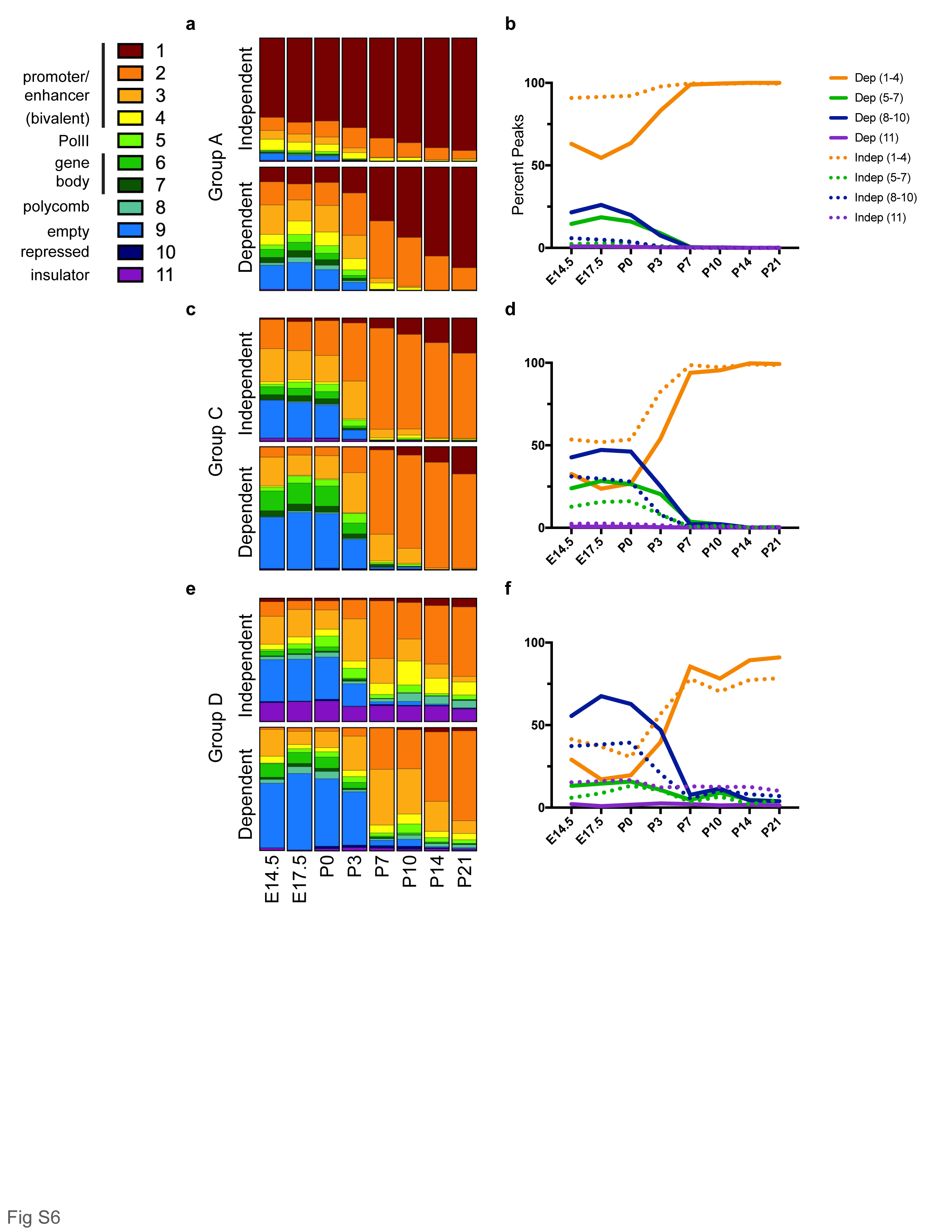

Supplement: Supplementary file 8 — Additional file 8: Fig. S6. Dependent and Independent sites show different chromatin state dynamics. (a, c, e) Stacked bargraphs represent proportion of Group A–D sites that are contained within each HMM-defined chromatin state at 8 developmental ages. (b, d, f) Quantification of data binned into HMM classes 1–4, 5–7, 8–10, and 11, for Dependent and Independent sites show different dynamics of reorganization over development. Legend defines basic classification of HMM classes [30]. [file 13072_2018_212_MOESM8_ESM.tif]

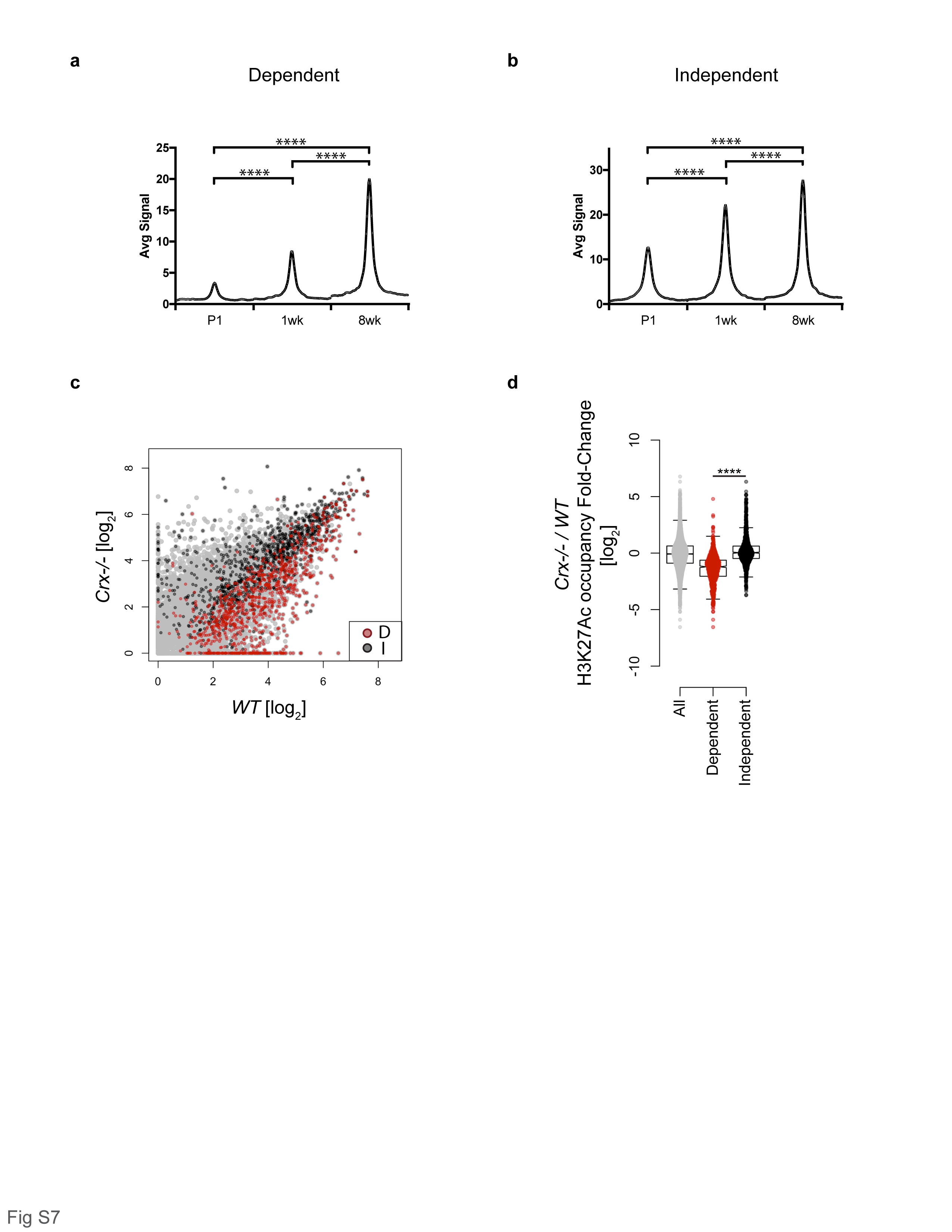

Supplement: Supplementary file 9 — Additional file 9: Fig. S7. CRX is required to activate Dependent Group C enhancer elements and remodel chromatin. Plots display mean (black line) and SEM (gray bars) of DNase I data presented in Fig. 5a for CRX Dependent (a) and Independent (b) sites. (Two-way ANOVA with Tukey multiple comparison testing; **** p<0.0001) (c) Scatterplot displays changes in H3K27Ac deposition at all peaks (light gray). Black and red denote the subset of H3K27Ac peaks that contain CRX Independent and Dependent Group C sites. (d) Quantification of the fold-change of H3K27Ac deposition in WT vs Crx-/-. (Wilcoxon Rank Sum Test; **** p < 2.2 × 10^−16^) [file 13072_2018_212_MOESM9_ESM.tif]

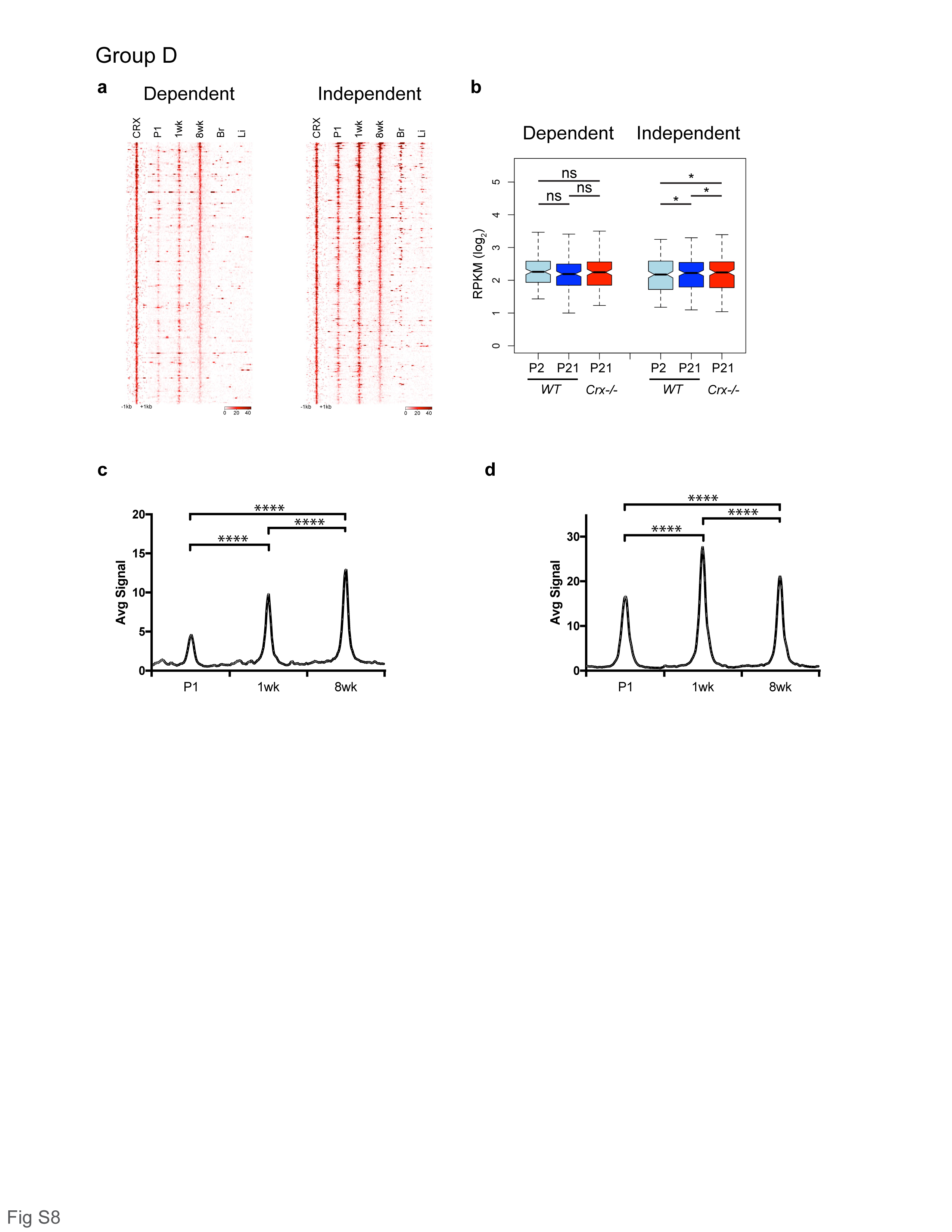

Supplement: Supplementary file 10 — Additional file 10: Fig. S8. CRX is required to activate Dependent Group D distal regulatory elements. a Plots display read density of DNase I experiments centered on CRX-binding site of Dependent and Independent Group D sites. b Analysis of RNA-seq of nearest gene to each peak, displayed as boxplot of normalized RPKM values at P2 and P21 in WT and P21 in Crx-/-. (Wilcoxon Rank Sum Test, Paired; *p < 0.05, **p < 2.2 × 10^−5^, ***p < 2.2 × 10^−10^, ****p < 2.2 × 10^−16^) Plots display mean (black line) and SEM (gray bars) of DNase I data above for CRX Dependent (c) and Independent (d) sites. (Two-way ANOVA with Tukey multiple comparison testing; ****p < 0.0001). [file 13072_2018_212_MOESM10_ESM.tif]

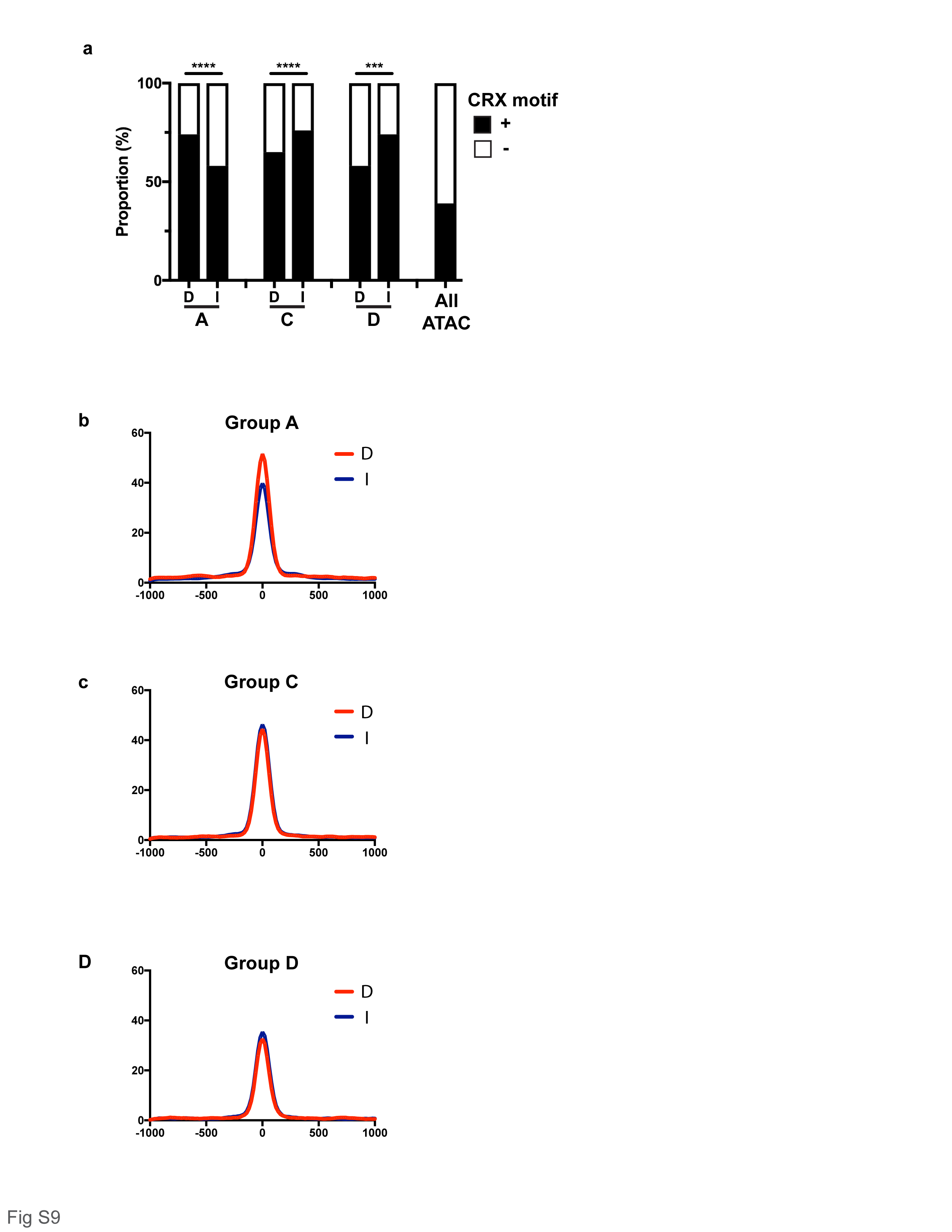

Supplement: Supplementary file 11 — Additional file 11: Fig. S9. Sites display different presence of CRX motif and binding. a Proportion of sites within each of the noted groups that contains the published CRX motif. (Fisher’s exact test; ***p = 0.0001, ****p < 0.0001) b–d Quantification of read depth of CRX ChIP-seq at Dependent (red) and Independent (blue) sites within the specified Groups. [file 13072_2018_212_MOESM11_ESM.tif]

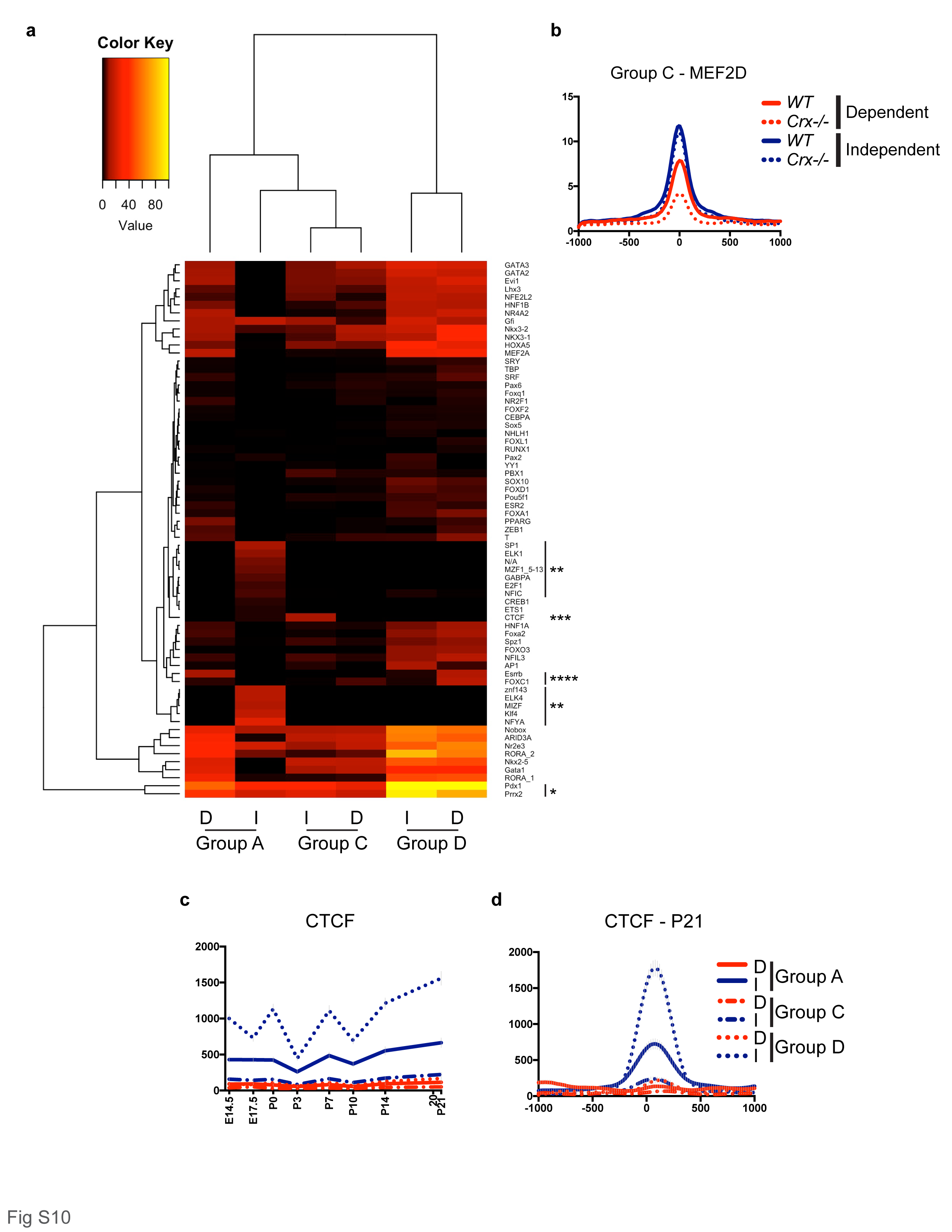

Supplement: Supplementary file 13 — Additional file 13: Fig. S10. TF motifs explain nature of Independent site activation in absence of CRX. a Heatmap shows unsupervised clustering of [−log10] transformed p-values representing the significance of representation of the noted TF motif within the set of sites. (* -****) represent TFs referenced in details in the text. b Quantification of MEF2D ChIP-seq data at Group C enhancer sites displays loss of signal specifically at Dependent sites (red) in the Crx−/−. Independent sites (blue) show no change in signal. Quantifications of CTCF binding over time (c maximum peak intensity over development and d relative to CRX bindings site at P21) display consistent signal at Group D Independent sites. [file 13072_2018_212_MOESM13_ESM.tif]
